# Exploring the Interplay of Explant Origin and Culture Density on Olive Micropropagation Efficiency

**DOI:** 10.3390/plants14081170

**Published:** 2025-04-09

**Authors:** Maroua Grira, Amal Rabaaoui, Els Prinsen, Stefaan Werbrouck

**Affiliations:** 1 Laboratory for Applied In Vitro Plant Biotechnology, Ghent University, Valentin Vaerwyckweg 1, 9000 Ghent, Belgium; 2 Integrated Molecular Plant Physiology Research, Department of Biology, University of Antwerp, Groenenborgerlaan 170, 2020 Antwerpen, Belgium

**Keywords:** olive, apical dominance, micropropagation, density, cytokinin, hormone migration, topophysis, shoot proliferation

## Abstract

Apical dominance and culture heterogeneity significantly limit the efficiency of olive micropropagation, hindering the rapid production of plantlets. This study explores how manipulating the explant origin (topophysis) and density can mitigate these challenges. Explants originating from apical and middle sections were cultivated at densities of 18, 24, and 30 explants per vessel. After 12 weeks, significant differences in the growth parameters were observed based on the explant origin and density. The middle-section explants exhibited superior shoot proliferation and node production, especially at higher densities. The callus weight also increased with the density, while the internode length remained relatively stable. Hormone analysis demonstrated the density-dependent spatial distribution pattern of aromatic and isoprenoid cytokinins. Notably, at higher densities, the aromatic free bases in the apical-section leaves showed migration toward the shoot apices, while this migration was less pronounced in the middle-section leaves. Isoprenoid cytokinins displayed complex distribution patterns, with free bases and O-glucosides often increasing toward the basal nodes. These findings demonstrate that optimizing the explant origin and density can effectively reduce apical dominance and enhance culture homogeneity in olive micropropagation. This approach offers a promising strategy for improving the micropropagation protocols for olive and potentially other woody plants, leading to more efficient and cost-effective production of high-quality plantlets for commercial use.

## 1. Introduction

Olive trees are culturally and economically central to the Mediterranean region, demonstrating resilience to climate change and producing valuable wild-olive (Oleaster (*Olea sylvestris*)) oil [1,2,3,4]. Despite its lower agronomic value [5], oleaster oil offers a nutritionally superior alternative to cultivated olive oil [3,6], exhibiting a high concentration of antioxidants [7] and phenolic compounds [8,9], with notable antibacterial [10], anti-inflammatory [11,12,13] and anti-tumoral properties [14,15]. The increasing global demand for olive oil [16,17], evidenced by a market valued at USD 18.6 billion in 2022 and projected to reach USD 30.2 billion by 2032 [18], necessitates the development of efficient olive production systems that effectively leverage olive genetic resources for adaptation [19,20]. Oleaster serves as a reservoir of genetic diversity for breeding programs targeting biotic and abiotic stress resistance [3,8]. Moreover, it is integral to reforestation efforts, as exemplified by its inclusion in the Porto Santo Island program since 2006 [21]. The vulnerability of some wild-olive species to extinction [21,22] underscores the critical importance of germplasm conservation through *O. sylvestris* [21,22]. Traditional propagation limitations render in vitro propagation a crucial strategy, despite the current lack of comprehensive micropropagation data for *O. sylvestris* [21].

While olive micropropagation is continually advancing [23], progress in in vitro culture remains constrained by the strong apical dominance, resulting in a limited multiplication factor and significant culture heterogeneity. Although apical meristem excision is not consistently effective [24], exogenous cytokinin (CK) application can stimulate axillary bud development, even in the presence of an intact apex [25]. To overcome this challenge, studies have explored the efficacy of various CKs, including zeatin (Z), benzyl-adenine (BA), kinetin (Kn), thidiazuron (TDZ), and meta-topolin (mT) [26], as well as CK-like substances like dikegulac [27]. Zeatin (Z) has shown particular promise in promoting axillary bud formation across diverse olive cultivars [26,28,29].

The exploration of CKs commenced with Miller in 1955 [30]. Its activity remained elusive until 1987, when Kaminek revealed the significant biological effects [31]. This discovery prompted an intensified investigations into CKs, as reported by Nisler [32]. Cytokinins (CKs), a class of phytohormones, represent chemical signals that control multiple developmental processes during the plant life cycle, including gametogenesis, root meristem specification, vascular development, shoot and root growth, meristem homeostasis, senescence, promotion of cell differentiation and more [33,34,35,36,37]. They also mediate responses to environmental cues such as light, stress and nutrient conditions. The presence of cytokinin (CK) in the culture medium, in addition to mineral nutrients, is crucial to inducing the proliferation or regeneration of certain species [38].

Cytokinins (CKs), adenine derivatives substituted at the N6 atom and classified as isoprenoid or aromatic [39], are key in vitro regulators of shoot meristem initiation, proliferation, apical dominance, senescence, rooting, and callus formation [40,41,42]. Isoprenoid and aromatic CKs exhibit distinct modes of action, activation, deactivation, and storage mechanisms [43,44]. Free bases represent the active CK forms [45], while ribosylation converts them to transport forms with reduced activity. O-glucosylation serves as a reversible deactivation mechanism via β-glucosidase-mediated formation of aglycons [46]. Conversely, N-glucosylation results in irreversible CK inactivation [47,48]. Cytokinin upregulation is influenced by various biotic and abiotic factors [49,50,51].

Topophysis, a concept initially mentioned by Molish in 1915, was further explored by William J. Robbins in 1964, who considered it a matter of somatic inheritance, and differentiated from cyclophysis in 1978 by Olesen [52]. It refers to the positional effect of the original plant material on subsequent growth and development [53,54]. This phenomenon had primarily been explored in relation to the rooting capacity in vivo [55,56,57,58] and scarcely in vitro [54,59,60]. In plants, particularly woody species, topophysis represents a significant challenge to vegetative propagation [61]. This positional effect can influence various aspects of plant growth and development. Understanding and overcoming the constraints imposed by topophysis are crucial to improving propagation techniques in horticulture and forestry.

Understanding the interplay between the explant origin, culture density, and cytokinin metabolism is crucial to optimizing olive micropropagation. Therefore, we hypothesize that manipulating the explant origin and increasing the culture density will alter the cytokinin metabolism and spatial distribution, ultimately improving the shoot proliferation and reducing the apical dominance effect on in vitro olive plants. Therefore, this research aims to investigate the effects of the explant origin (apical vs. middle sections) and explant density on the following: (1) morphological parameters such as the number of shoots, the length of shoots (cm), the number of nodes per plant, the weight of the callus (g) and the length of the internodes (cm); and (2) the spatial distribution of aromatic and isoprenoid cytokinins in the leaves and stems of olive shoots in vitro. Furthermore, we explore the correlation between the cytokinin distribution patterns and the observed morphological changes. By elucidating these interactions through spatial analysis of cytokinin metabolites and correlating them with morphological data, this research aims to provide a deeper understanding of olive micropropagation and contribute to more effective strategies for managing in vitro culture variability.

## 2. Results

### 2.1. Morphological Parameters

**Figure 1:**
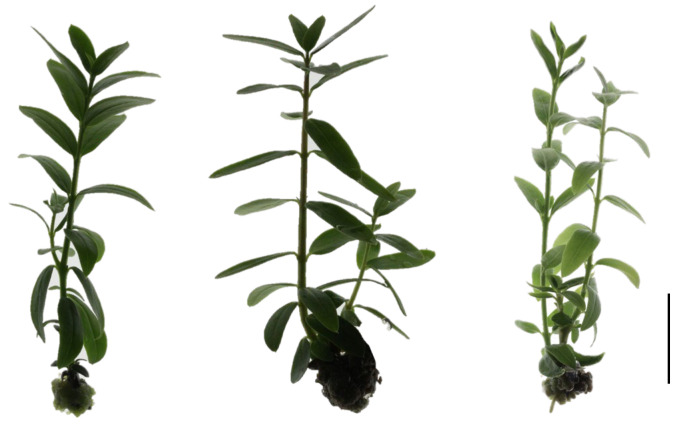
In vitro oleaster plant developed from a shoot originating from the apical section (SAS) cultivated under D18 (**left**); D24 (**middle**) and D30 (**right**) (scale bar is 0.5 cm).

In shoots originating from the apical section (SAS), the analysis of the effects of the explant density on various morphological parameters reveals several key trends (Figure 1, Table 1). Due to the regular removal of callus during the subculture, the observed shoots are presumed to have originated from axillary buds on the stem explant. As the explant density increases from 18 to 30, the number of shoots rises from 1.5 to 2.1. However, the length of the shoots slightly decreases, showing a trend from 2.1 cm at 18 explants to 1.9 cm at 30 explants. The total number of nodes per plant significantly rises from 7.2 to 11.3 with the increase in the density. The callus weight also increases with the density from 0.18 g to 0.26 g. Conversely, the internode length slightly decreases with the increase in the density between 0.4 and 0.34.

**Figure 2:**
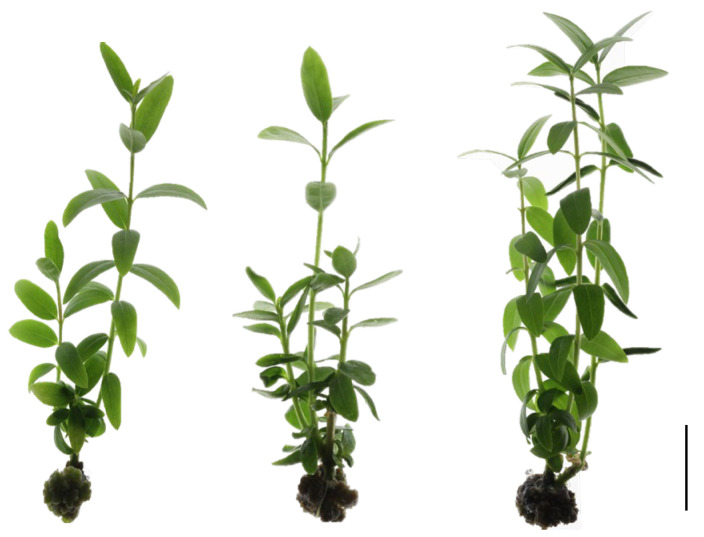
In vitro oleaster plant developed from a shoot originating from the middle section (SMS) cultivated under D18 (**left**); D24 (**middle**) and D30 (**right**) (scale bar is 0.5 cm).

In shoots originating from the middle section (SMS), the dataset shows more pronounced effects of the explant density on several morphological parameters (Figure 2, Table 2). Given the experimental design, which included regular callus removal, the observed shoots developed from axillary buds present on the stem explant. As the density increases from 18 to 30 explants per vessel, the number of shoots increases significantly from 2.2 to 3.4. The length of the shoots shows a slight increase from 1.5 cm to 1.7 cm. The total number of nodes per plant increases dramatically from 10 to 17 as the density increases. The callus weight shows a substantial increase from 0.16 g to 0.38 g with increasing density. Interestingly, the internode length remains constant at 0.3 cm across all the densities.

### 2.2. Cytokinin Analysis

The cytokinin analysis results are presented using heat map figures. The absolute concentrations in pmol/g DW of each conjugate and each group of both aromatic (AR) and isoprenoid (ISO) cytokinins (CKs) are detailed in the Supplementary Materials data file.

#### 2.2.1. Aromatic Cytokinins

**Figure 3:**
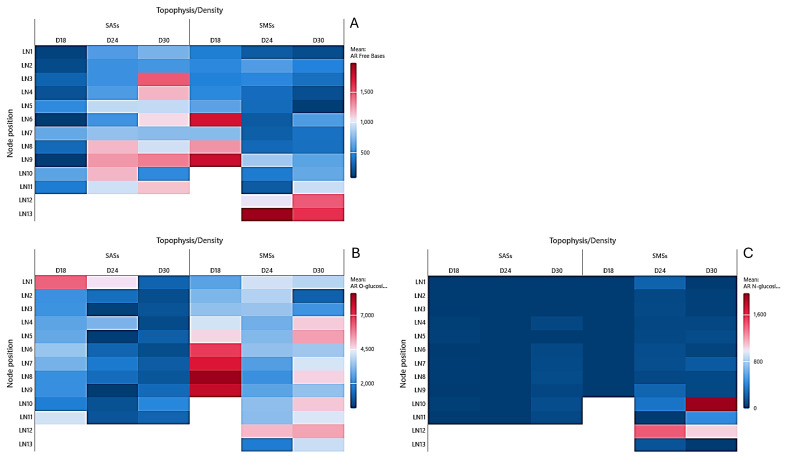
Heat map of the aromatic cytokinins in the leaves of in vitro oleaster, showing the (**A**) free bases, (**B**) O-glucosides, and (**C**) N-glucosides. Shoots originating from the apical part (SAS) and middle part (SMS) under varying densities: D18 (18 explants/vessel), D24 (24 explants/vessel), and D30 (30 explants/vessel). LN denotes leaves of the node, with numbers 1–13 indicating the node position from top to bottom.

**Figure 4:**
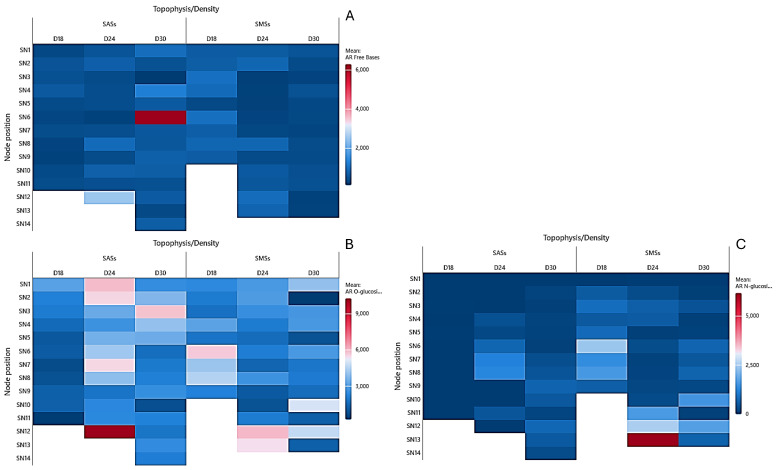
Heat map of the aromatic cytokinins in the stems of in vitro oleaster, showing the (**A**) free bases, (**B**) O-glucosides, and (**C**) N-glucosides. Shoots originating from the apical part (SAS) and middle part (SMS) under varying densities: D18 (18 explants/vessel), D24 (24 explants/vessel), and D30 (30 explants/vessel). SN denotes stems of the node, with numbers 1–14 indicating the node position from top to bottom.

The AR free base concentrations in the SAS leaves exhibited a density-dependent distribution pattern. As illustrated in Figure 3A, the concentrations were the lowest at D18, reaching a maximum of 647 pmol/g DW at the base. The increase in the explant’s density to D24 resulted in a twofold elevation of the AR free bases, with a shift toward the shoot’s midsection. At the highest density (D30), the AR free bases demonstrated further migration to higher apical nodes, reaching a maximum of 1.476 pmol/g DW in the leaf tissue of N3. In the SMS leaves (Figure 4A), the AR free base migration to apical regions was less pronounced. The highest concentrations were consistently observed in the basal nodes, with values of 1.803, 1.978, and 1.615 pmol/g DW at D18, D24, and D30, respectively.

The AR O-glucoside concentrations in the SAS leaves displayed an inverse correlation with the density. Figure 3B illustrates that in leaves, the AR O-glucoside concentrations decreased as density increased, with ranges of 6.245–1.682, 4.496–172, and 1.876–505 pmol/g DW in D18, D24, and D30, respectively. The SMS leaves exhibited the apical migration of AR O-glucosides, with a decreasing trend as the density increased. D18 accumulated the highest values (2.538–8.635 pmol/g DW), followed by D24 (2.113–5.019 pmol/g DW) and D30 (1.091–5.374 pmol/g DW).

The AR N-glucosides showed minimal variation across densities in the SAS leaves, as depicted in Figure 3C. These compounds were absent in the SMS leaves at D18 and SAS leaves at D24. At the highest density (D30), the AR N-glucoside concentrations in the SAS leaf tissue did not exceed 89 pmol/g DW. However, in the SMS leaf tissue, they reached 1.050 pmol/g DW.

In the stem tissue, the AR free bases demonstrated reduced apical migration compared to the leaf tissue as the density increased (Figure 4A). At higher densities, the AR free bases were more abundant in the SAS stems (6.294 pmol/g DW at D30 and 2.466 pmol/g DW at D24) compared to the SMS stems (484 pmol/g DW at D30 and 880 pmol/g DW at D24). However, at D18, the concentrations were higher in the SMS stem tissue than in the SAS (936 and 578 pmol/g DW, respectively).

The AR O-glucosides in the stem tissue were more concentrated at the shoot base (Figure 4B). Across all the nodes, the AR O-glucoside values were more abundant in the SMS stems; however, they reached higher maximum values in the SAS (3.033, 10.239, 5.818 pmol/g DW) than in the SMS (5.697, 5.976, 5.004 pmol/g DW).

The AR N-glucosides were not detected in the SAS leaves and stems at D18 (Figure 4C). However, they were more prevalent in the stem tissue of both the SMS and SAS at D18 and D24, with concentrations ranging from 492 to 2.359 pmol/g DW for the SMS and 202 to 1.240 pmol/g DW for the SAS. At the highest density (D30), the AR N-glucoside concentrations in the SAS stem tissue varied from 74 to 713 pmol/g DW.

#### 2.2.2. Isoprenoid Cytokinins

**Figure 5:**
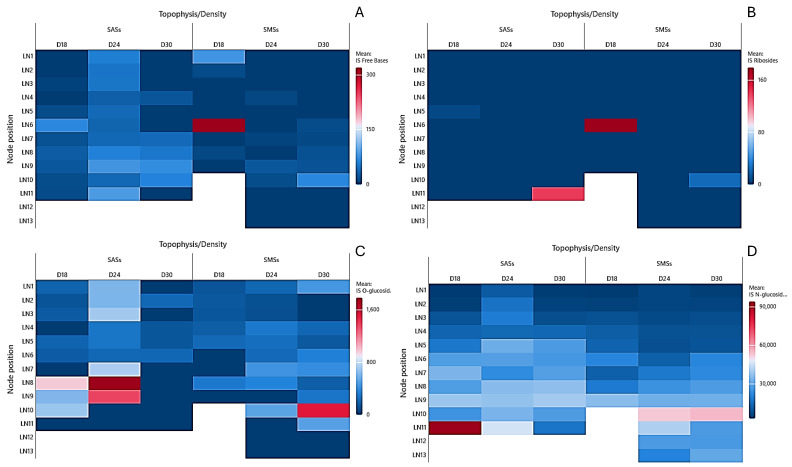
Heat map of the isoprenoid cytokinins in the leaves of in vitro oleaster, showing the (**A**) free bases, (**B**) ribosides, (**C**) O-glucosides, and (**D**) N-glucosides. Shoots originating from the apical part (SAS) and middle part (SMS) under varying densities: D18 (18 explants/vessel), D24 (24 explants/vessel), and D30 (30 explants/vessel). LN denotes leaves of the node, with numbers 1–13 indicating the node position from top to bottom.

The ISO free bases in the SAS leaves were absent at the apex of D18 and D30, but present in all the nodes of D24, peaking to 83 pmol/g DW in the leaves of the basal node (Figure 5A). In the SMS leaves, they were not detected in most nodes of D24 and D30. Remarkably, at the lowest density, the concentrations were higher, reaching 316 pmol/g DW in the leaves of N6.

In the leaf tissue of the SAS, the ISO ribosides were detected only at the basal node of the SAS (N11) at D30, reaching 141 pmol/g DW. In the SMS leaves, they were present only in N6 of D24, reaching 180 pmol/g DW (Figure 5B).

The ISO O-glucoside in the SAS leaves were more concentrated in the apical node of D30 up to 211 pmol/g DW (Figure 5C). In contrast, D18 and D24 showed higher concentrations in the basal node (N8) of up to 967 and 1.779 pmol/g DW, respectively.

The ISO N-glucosides in the SAS leaves reached their maximum concentrations in N8 of D18 and D24 (972 and 1.788 pmol/g DW, respectively) (Figure 5D). In contrast, the SMS leaves displayed no clear trend, with the values fluctuating between 0 and 1.500 pmol/g DW across all the densities.

**Figure 6:**
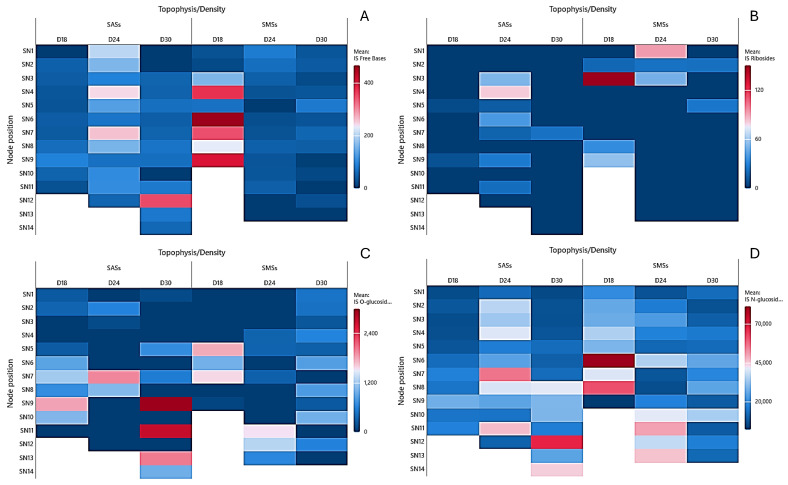
Heat map of the isoprenoid cytokinins in the stems of in vitro oleaster, showing the (**A**) free bases, (**B**) ribosides, (**C**) O-glucosides, and (**D**) N-glucosides. Shoots originating from the apical part (SAS) and middle part (SMS) under varying densities: D18 (18 explants/vessel), D24 (24 explants/vessel), and D30 (30 explants/vessel). SN denotes stems of the node, with numbers 1–13 indicating the node position from top to bottom.

In the SAS stems, the ISO free base concentrations varied at D18 without a clear trend (26–87 pmol/g DW) (Figure 6A). However, the concentrations increased toward the base of the shoot at D24, ranging from 51 to 261 pmol/g DW. Furthermore, at the highest density, the concentrations reached a notable peak of 355 pmol/g DW in N12. In the SMS stems, the ISO free bases were highest at the lowest density (26 to 500 pmol/g DW) and decreased at D24 and D30 (0 to 80 pmol/g DW).

The ISO ribosides were present in trace amounts in the SAS stems at D18 and D30 but more abundant at D24, up to 82 pmol/g DW. In the SMS stems, the ISO ribosides were concentrated toward the shoot apex, reaching up to 151 pmol/g DW at D18 (Figure 6B).

The ISO O-glucoside in the SAS stems varied across densities (Figure 6C). At D18, they ranged from 236 to 1.843 pmol/g DW, with some absence in certain nodes and an increasing trend toward the base. Conversely, at D24, they were detected in only a few nodes, with a maximum at N6 (1.977 pmol/g DW). At the highest density, the ISO O-glucosides were more abundant, ranging from 120 to 3.007 pmol/g DW, with an increasing trend toward the base. In the SMS stems, the ISO O-glucoside profiles differed from those in the leaf tissue (Figure 6C). At D18, they were quantified only in N4 to N6 (1.791, 962, and 1.575 pmol/g DW, respectively). At D24, the ISO O-glucosides were more abundant at the base (N11 to N13), reaching 1.526 pmol/g DW in N11. However, at the apex of the shoots, they were quantified only in N4, N5, and N7 (678, 319, and 298 pmol/g DW, respectively). At D30, they varied between 220 and 1531 pmol/g DW and were present in almost all the nodes.

In the SAS stems, the ISO N-glucosides were significantly higher at the base than at the apex at D18, ranging from 5.128 to 16.913 pmol/g DW (Figure 6D) With increasing density, the ISO N-glucosides increased throughout the nodes, varying between 9.853 and 56.673 pmol/g DW. Furthermore, the values continued to increase at D30, ranging from 4.656 to 67.567 pmol/g DW with an increasing trend toward the base of the shoot.

In the SMS stems, the ISO N-glucosides increased toward the base of the shoot at D18 and D24, reaching maximum values of 81.241 and 50.460 pmol/g DW, respectively. However, at the highest density, the values continued to increase toward the basal section, reaching a maximum of 33.870 pmol/g DW in N10.

## 3. Discussion

### 3.1. Morphological Parameters

Our study on the effects of the explant density on oleaster (*Olea europaea* subsp. *sylvestris*) shoots, derived from apical and middle sections, provides valuable insights into the combined effects of density and topophysis on in vitro growth, particularly for optimizing the micropropagation protocols for woody trees and olive plants. The differentiation of the shoots according to their topophysical origin showed that under the same culture density, the middle-section-originating shoots were more productive compared to the apical-section-originating shoots in terms of the number of shoots and nodes. Furthermore, the apical dominance was less dominant in the shoots originating from the middle section compared to the shoots originating from the apical sections.

Consistent with several studies, our findings demonstrated a strong positive correlation between explant density and shoot proliferation. For instance, similar results have been reported in banana [62], suggesting that increasing the density is effective in enhancing the number of shoots and nodes per shoot. In Moris pineapple, comparable results were also reported by Hamad [63], where increasing the explant density (up to five explants per 10 mL of culture medium) increased the shoot formation. Furthermore, Aycan et al. [64] observed that reducing the space between explants (effectively increasing the density) improved the regeneration percentage, shoot number per plant and per pot in *Lathyrus chrysanthus* Boiss. These findings align with our results, which show that increasing densities increased the number of shoots per plant.

Additionally, Mohamed et al. [65] reported that the percentage of sprouted nodes in potato was highest at the highest density, suggesting that higher densities can enhance specific growth parameters. This observation corresponds to our observation of an increased total number of nodes at higher densities, indicating a positive response to the culture environment. Similarly, Yildiz et al. [66] reported that increasing the density inside the culture container improved the shoot number and the total regenerated number of shoots on *Linum usitatissium*, further supporting our findings.

Remarkably, our observation of increased callus formation at higher densities in both the SAS (shoots originating from the apical section) and SMS (shoots originating from the middle section) might be related to enhanced cell-to-cell contact or accumulation of growth regulators at higher densities.

However, it is important to acknowledge that the effects of increased explant densities on growth parameters have been reported to vary across studies, with some research indicating negative impacts. For instance, Nguyen and Kozai [67] and El Boullani et al. [68] observed that the multiplication rate of artichoke explants decreased with increasing density. Mohamed and Shabaan [65] attributed this negative correlation to competition among plantlets for nutrients. Similarly, Chun et al. [69] proposed the hypothesis of plant competition in their study on *Populus alba* × *P. grandidentata*, where they found that increasing the explant number per vessel reduced the shoot number and fresh weight, presumably due to nutrient competition. Yildiz [70] also revealed that increasing the shoot density while maintaining the same amount of culture medium had a negative effect on the morphological growth of in vitro flax. However, it is crucial to note that our experimental design maintained a constant medium-to-plant ratio across treatments, thereby eliminating nutrient competition as a primary factor influencing the growth patterns. Consequently, this approach negates the hypothesis of a negative correlation due to nutrient competition between explants in our study.

Interestingly, the highest density of 30 explants per vessel identified in our study for maximizing the propagation efficiency aligns with findings by Tábori et al. [71] in potato. They reported that increasing the culture density up to 30 and 40 explants per jar increased the shoot length and the yield and shortened the cycle of tuber formation compared to a lower density (10 explants per jar).

The best results in terms of the number of nodes and number of shoots were observed in shoots originating from the middle section compared to shoots originating from the apical section. These results, if taken into consideration, could potentially lead to more efficient and targeted micropropagation strategies across various plant species and explant types.

### 3.2. Cytokinin Analysis

Abiotic stress and high-density culture impose similar challenges on plants, triggering physiological and biochemical adaptations essential for survival. These shared responses underscore the critical role of cytokinin modulation and adaptive growth strategies in enabling plants to cope with diverse environmental pressures. The effectiveness of these mechanisms depends on several factors, such as the plant’s phenological stage, the intensity and duration of stress, and the specific tissues or organs involved in compensatory processes [72]. Consistent with Cortleven et al. [73] and Mughal et al. [74], our findings demonstrate that plants modulate their CK levels in response to environmental challenges. Importantly, our study highlights the critical role of explant topophysis in determining CKs’ dynamics and plant growth response under varying culture densities. We observed distinct migration patterns of CK types within the plant that were strongly influenced by the explant origin and culture densities. The differentiation of shoots according to their topophysical origin showed that AR free bases migrated to the base of the shoots in the leaves of middle-section-derived shoots (SMSs) compared to apical-section-derived shoots (SASs). Similar trends were observed for AR O- and N-glucosides. In the stems, AR O- and N-glucosides followed comparable patterns. Notably, increasing the explant density induced divergent CK migration: aromatic free bases and aromatic O-glucosides accumulated in the shoot apices, while isoprenoid free bases and isoprenoid N-glucosides migrated toward the shoot base.

The increase in the explant density led to a notable elevation in the total CK content: 29% and 6% in the SAS and 54% and 24% in the SMS for the D30 and D24 treatments, respectively. This elevation may serve as a defense mechanism against indirect stress caused by increased density within the confined culture vessel volume, likely due to elevated temperature and humidity. These observations align with studies demonstrating abiotic-stress-induced increases in endogenous CK levels in maize [75,76]. The growth of in vitro plants is significantly affected by the internal microenvironment of the culture vessels, which remains challenging to adjust [77]. Jackson et al. [78] reported that low ventilation in *Ficus lyrata* Warb., *Gerbera jamesonii* Bolus, and *Solanum tuberosum* L. cv. Red Craig’s vessels caused ethylene and CO_2_ accumulation compared to ventilated vessels. Similarly, Kumar et al. [79] observed enhanced *Pinus radiata* tissue differentiation and early initiation phases under sealed conditions with ethylene/CO_2_ accumulation, correlating with our findings of improved wild-olive growth in non-ventilated, high-density cultures. Heuser and Heinemann [80] documented analogous effects in *Rhododendron*, where sealed vessels increased the shoot number and fresh weight due to the accumulation of ethylene.

Lentini et al. [81] compared *Brassica campestris* cultures in sealed versus ventilated containers, finding higher ethylene accumulation (manifested by leaf epinasty) and condensation in sealed systems, which is in correspondence with our results, where the figures showed leaf epinasty, which could be explained by the accumulation of ethylene inside the closed culture vessel. Vanderschaeghe and Debergh [82] and Chen [83] further reported that light irradiance elevates internal culture vessel temperatures, while Majada et al. [84] investigated closed versus ventilated microclimates in carnation cultures. Chen and Chen [77] emphasized the limited adjustability of this critical microenvironment.

The elevated CK content observed in the higher density treatments correlates with increased ethylene production, as reported by Fatma et al. [85]. This relationship manifested in our study through leaf epinasty (downward bending) in the D24 and D30 treatments (Figure 2 and Figure 3), a characteristic response to high ethylene levels [86]. These findings align with those of Love et al. [87], who demonstrated that endogenous ethylene stimulates stem meristematic growth in ethylene-sensitive poplar mutants cultured in vitro. The combination of this ethylene-induced response and the specific distribution of cytokinin conjugates made the highest density treatment the most productive for in vitro culture of oleaster. An additional benefit of the increased density was the improved culture homogeneity.

Our findings both support and challenge the hypothesis of Zwack and Rashotte [88] regarding reduced long-distance CK transport under abiotic stress. In the stems of shoots originating from the middle sections (SMS), increasing the explant density decreased the aromatic and isoprenoid cytokinin contents, aligning with their hypothesis. However, shoots from the apical sections (SAS) exhibited the opposite trend: higher density enhanced long-distance aromatic and isoprenoid cytokinin transport, evidenced by their increased amounts and their apical migration. This contrast points out the critical influence of the explant topophysical origin on the CK dynamics under stress. The differing responses between the SMS and SAS suggest that apical tissues may possess unique physiological properties or stress response mechanisms. Therefore, it is essential to consider the explant origin when studying hormone responses, as it can significantly impact the results, even within the same species.

Previous studies have reported conflicting results regarding the CK content in response to stress, with some indicating increases [89,90,91] and others showing decreases [92,93,94]. These inconsistencies reflect the complexity of plant stress responses. Our study adds to this dialogue by demonstrating that different tissue origins can exhibit opposing CK responses to stress, emphasizing the importance of tissue-specific analysis in future research to better understand the CK dynamics under in vitro culture conditions.

Our findings on isoprenoid conjugates in in vitro oleaster differ somewhat from those reported by Tekaya et al. [95] for in vitro olive (cv Chetoui). While they found tZR and iP9G to be predominant, our study identified DHZN7G as the primary isoprenoid conjugate, followed by iP9G. Interestingly, we observed significant levels of ZN7G, DHZ9G, and iP7G, which Tekaya et al. [95] suggested as stress-related “priming fingerprints”. This correlation implies that increasing the explant density may induce stress conditions, potentially triggering enhanced multiplication and improved shoot homogeneity. These differences in the cytokinin profiles between closely related species highlight the complexity of the hormone dynamics in in vitro cultures and underscore the need for species-specific optimization of culture conditions.

## 4. Materials and Methods

**Figure 7:**
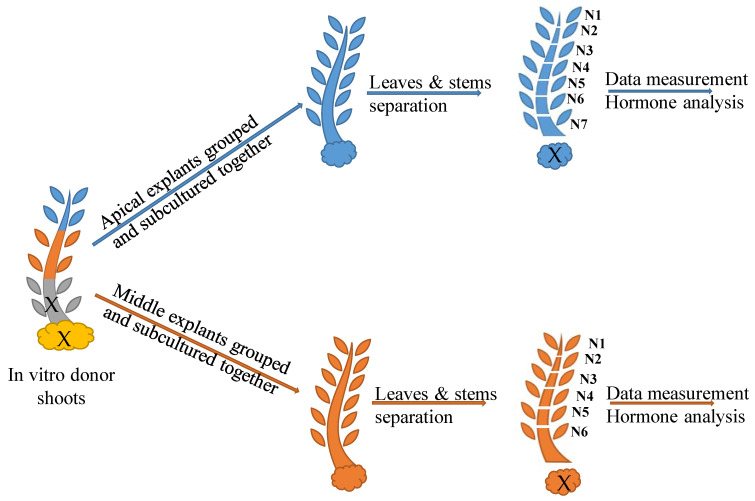
Experimental design and sample identification of the apical and middle topophysical sections. Apical section in blue and middle section in orange. X means discarded.

**Figure 8:**
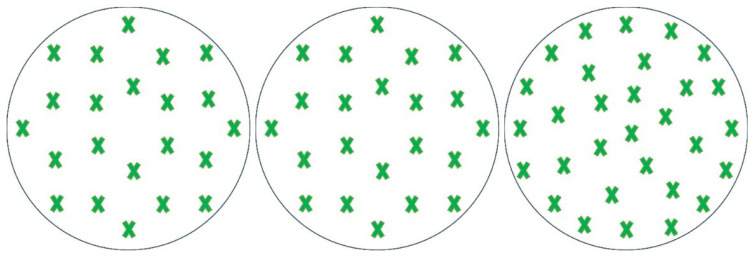
Experimental design and disposition of plants according to a density of 18 (**left**), 24 (**middle**) and 30 (**right**) explants per culture vessel.

Explants were taken from two different topophysical sections of in vitro donor shoots of oleaster plants: the apical and the middle shoot section, as explained in Figure 7. Each explant had two nodes and measured approximately 1.5 cm. They were grouped according to the same topophysical origin and cultivated under different densities inside the culture vessel: 18 explants (D18), 24 explants (D24) and 30 explants (D30) (Figure 8). The explants were cultivated in 720 mL glass vessels with screw-polycarbonate lids, on Rugini olive medium (Duchefa Biochemie, Haarlem, The Netherlands) supplemented with 30 g/L sucrose, 5 µM Z (Duchefa Biochemie, Haarlem, The Netherlands) and 7 g/L plant agar (Duchefa Biochemie, Haarlem, The Netherlands). The pH was adjusted to 5.8 before autoclaving. The amount of the culture medium was proportionally increased with the increase in the density, keeping it at 8 mL of medium per explant.

The cultures were maintained in a growth chamber at 26 ± 2 °C under a 16 h photoperiod. Cool fluorescent light was provided by a PHILIPS master TLD 36 W 830 Reflex ECO (Philips, Ghent, Belgium) (40 μmol m^−2^ S^−1^ PAR). After 12 weeks of subculture, the following parameters were recorded: number of shoots, length of shoot, number of nodes per plant, callus weight and the length of the internodes. At the end of the multiplication cycle, the leaves and stem of each node were separated and numbered. The node numbering followed a descendant echelon: N1 represented the apical node, N2 the second apical node and so on (Figure 8). This numbering system was consistent for shoots originating from the apical and the middle sections. The nodes and leaves were analyzed for aromatics (ARCKs) and isoprenoids (ISCKs).

Divided into four groups: free bases, ribosides, O- and N-glucosides. The AR free bases included BA (6-benzylaminopurine), pT (para-topolin), mT (meta-topolin) and MeoT (ortho-methoxy-topolin). The AR O-glucosides included mTOG (meta-topolin-O-glucoside), pTOG (para-topolin-O-glucoside), mTROG (meta-topolin-riboside-O-glucoside). The AR N-glucosides included BA7G (6-benzyladenine-7-glucoside), mT9G (meta-topolin-9-glucoside), mT7G (meta-topolin-7-glucoside), MemT9G (meta-methoxy-topolin-9-glucoside) and oT9G (ortho-topolin-9-glucoside). The ISO free bases included tZ (trans-zeatin) and iPA (6-isopentenyladenosine). The ISO ribosides included DHZR (dihydrozeatin riboside) and tZR (trans-zeatin riboside). The ISO O-glucosides included DHZOG (dihydrozeatin O-glucoside), ZOG (zeatin O-glucoside), ZROG (zeatin riboside O-glucoside) and DHZROG (dihydrozeatin riboside O-glucoside), and the ISO N-glucosides included DHZ7G (dihydrozeatin 7-glucoside), ZN7G (zeatin 7-glucoside), DHZN9G (dihydrozeatin 9-glucoside), ZN9G (zeatin 9-glucoside), iP7G (6-isopentenyladenosine 7-glucoside) and iP9G (6-isopentenyladenosine 9-glucoside).

The cytokinin analysis was performed as detailed in Grira et al. [96]. Here, 25–300 mg of lyophilized leaves and stem material was homogenized and sonicated, with overnight extraction at −20 °C. The ISCKs were analyzed using a Waters ACQUITY UPLC (Sigma-Aldrich, Hoeilaart, Belgium) with ES(+) TQD Tandem Quadrupole MS/MS in MRM mode. The separation used a BEH C18 Column (Sigma-Aldrich, Hoeilaart, Belgium) with pre-column. A gradient of ammonium acetate (molecular biology grade 7.5 M, Merck Sigma-Aldrich, Hoeilaart, Belgium) in water and methanol (HiPerSolv CHROMANORM^®^; VWR, Leuven, Belgium) and B methanol (MeOH) (HiPerSolv CHROMANORM^®^; VWR, Leuven, Belgium) was applied. Cytokinins were identified by comparing the fragmentation and retention times with pure standards (Olchemim, Olomouc, Czech Republic).

The ARCKs were analyzed using a separate UPLC-TQD MS/MS (Sigma-Aldrich, Hoeilaart, Belgium) run, employing the same guard and analytical column as for the ISCKs. The chromatographic conditions were as described: 95:5 A:B with A 0.1 M ammonium acetate (diluted from molecular biology grade, 7.5 M, Merck Sigma-Aldrich, Hoeilaart, Belgium) in water (HiPerSolv CHROMANORM^®^; VWR, Leuven, Belgium) and B methanol (HiPerSolv CHROMANORM^®^; VWR, Leuven, Belgium), 99.9:0.1 A:B to 95:5 A:B to 72:28 A:B for 5 min, isocratic 72:28 A:B for 0.5 min, linear gradient 72:28 A:B to 0.1:99.9 A:B for 30 s, flow 0.4 µL min^−1^, column temperature 48 °C, injection volume 6 µL. O-glucoside pure standards are not available, so these derivates were characterized and qualified using β-glucosidase treatment (5 U, from almonds, 2 U/mg, Merck Sigma-Aldrich, Hoeilaart, Belgium) on half of the samples following Werbrouck et al. [97].

All the experiments were conducted using a randomized complete block design with three blocks. Each block contained five replicates of each treatment combination (2 shoot positions × 3 densities). Each replicate consisted of 18, 24, or 30 shoots for D18, D24, and D30 treatments, respectively. Data were analyzed using the Kruskal–Wallis test, at *p* ≤ 0.05. Statistical analyses were conducted using IBM SPSS Statistics for Windows, version 29 (IBM Corp., Armonk, NY, USA). A significance level of α = 0.05 was employed throughout the analyses. The non-parametric Kruskal–Wallis test was employed to evaluate the group differences for the morphological and hormone analysis results.

## 5. Conclusions

Our study provides valuable insights into the complex interplay between the explant origin, culture density, and olive micropropagation efficiency. While there is no universally preferable explant origin across all olive varieties, our findings demonstrate that the choice of explant source significantly influences the micropropagation outcomes. We observed that shoots originating from the middle section, when cultivated at the highest density of 30 explants per pot, maximized the propagation efficiency. However, the apical-section shoots also exhibited improved growth parameters at higher densities, potentially mitigating the apical dominance issues commonly encountered in olive micropropagation. The optimal explant origin may vary depending on the specific olive cultivar and micropropagation objectives. Our results emphasize the importance of separating microshoots by origin during the in vitro multiplication phase to optimize the efficiency. This approach allows for tailored cultivation conditions that account for the distinct responses of apical- and middle-section explants to increasing density. In conclusion, while our research does not identify a universally preferable explant origin, it underscores the importance of considering the explant source as a critical factor in olive micropropagation protocols. Future studies should continue to explore the interactions between the explant origin, culture conditions, and specific olive cultivars to further refine and optimize the micropropagation techniques for this economically important species.

## Figures and Tables

**Table 1:** Morphological parameters of oleaster developed from shoots originating from the apical section (SAS) cultivated under D18, D24 and D30.

	Number of Shoots	Length of Shoots (cm)	Number of Nodes/Plant	Weight of Callus (g)	Length of Internodes (cm)
D18	1.5 ± 0.2 b	2.1 ± 0.08 a	7.2 ± 0.2 b	0.18 ± 0.01 b	0.4 ± 0.09 a
D24	2.0 ± 0.1 a	2 ± 0.05 a	10.4 ± 0.01 a	0.22 ± 0.01 ab	0.35 ± 0.1 b
D30	2.1 ± 0.1 a	1.9 ± 0.02 a	11.3 ± 0.01 a	0.26 ± 0.01 a	0.34 ± 0.09 b

Means followed by the same letter are not significantly different (*p* ≤ 0.05; Kruskal–Wallis test).

**Table 2:** Morphological parameters of oleaster developed from shoots originating from the middle section (SMS) cultivated under D18, D24 and D30.

	Number of Shoots	Length of Shoots (cm)	Number of Nodes/Plant	Weight of Callus (g)	Length of Internodes (cm)
D18	2.2 ± 0.02 b	1.5 ± 0.05 b	10.7 ± 1.1 c	0.16 ± 0.02 c	0.3 ± 0.08 a
D24	3 ± 0.01 a	1.6 ± 0.06 ab	14.5 ± 1.1 b	0.27 ± 0.025 b	0.3 ± 0.06 a
D30	3.5 ± 0.01 a	1.7 ± 0.01 a	17.3 ± 0.9 a	0.38 ± 0.03 a	0.3 ± 0.07 a

Means followed by the same letter are not significantly different (*p* ≤ 0.05; Kruskal–Wallis test).

## Data Availability

Data are contained within the article and Supplementary Materials.

## Supplementary Materials

The following supporting information can be downloaded at https://www.mdpi.com/article/10.3390/plants14081170/s1, Table S1: Aromatic cytokinin conjugates (BA: benzyl-aminopurine, mT: meta-topolin, MeoT: ortho-methoxy-topolin, mTOG: meta-topolin-O-glucoside, mT7G: meta-topolin-7-glucoside, pTOG: para-topolin-O-glucoside, mTROG: meta-topolin-riboside-O-glucoside) in shoots originated from the apical section (SASs) of in vitro Oleaster, cultivated at density of 18 (D18). LN refers to leaves of node and the numbers from 1 to 11 refer to the number of the node starting from 1 the top node; Table S2: Aromatic cytokinin conjugates (BA: benzyl-aminopurine, MeoT: ortho-methoxy-topolin, mTOG: meta-topolin-O-glucoside, mT7G: meta-topolin-7-glucoside, pTOG: para-topolin-O-glucoside, mTROG: meta-topolin-riboside-O-glucoside) in shoots originated from the apical section (SASs) of in vitro Oleaster, cultivated at density of 18 (D18). SN refers to stems of node and the numbers from 1 to 11 refer to the number of the node starting from 1 the top node; Table S3: Aromatic cytokinin conjugates (BA: benzyl-aminopurine, mT: meta-topolin, MeoT: ortho-methoxy-topolin, mTOG: meta-topolin-O-glucoside, pTOG: para-topolin-O-glucoside, mTROG: meta-topolin-riboside-O-glucoside) in shoots originated from the middle section (SMSs) of in vitro Oleaster, cultivated at density of 18 (D18). LN refers to leaves of node and the numbers from 1 to 9 refer to the number of the node starting from 1 the top node; Table S4: Aromatic cytokinin conjugates (BA: benzyl-aminopurine, MeoT: ortho-methoxy-topolin, mT9G: meta-topolin-9-glucoside, mTOG: meta-topolin-O-glucoside, pTOG: para-topolin-O-glucoside, mTROG: meta-topolin-riboside-O-glucoside, MemT9G: meta-methoxy-topolin-9-glucoside) in shoots originated from the middle section (SMSs) of in vitro Oleaster, cultivated at density of 18 (D18). SN refers to stems of node and the numbers from 1 to 9 refer to the number of the node starting from 1 the top node; Table S5: Aromatic cytokinin conjugates (BA: benzyl-aminopurine, pT: para-topolin, mT: meta-topolin, MeoT: ortho-methoxy-topolin, mTOG: meta-topolin-O-glucoside, pTOG: para-topolin-O-glucoside) in shoots originated from the apical section (SASs) of in vitro Oleaster, cultivated at density of 24 (D24). LN refers to leaves of node and the numbers from 1 to 11 refer to the number of the node starting from 1 the top node; Table S6: Aromatic cytokinin conjugates (BA: benzyl-aminopurine, pT: para-topolin, mT: meta-topolin MeoT: ortho-methoxy-topolin, BA7G: benzyladenine-7-glucoside, mT9G: meta-topolin-9-glucoside, mTOG: meta-topolin-O-glucoside, pTOG: para-topolin-O-glucoside, mTROG: meta-topolin-riboside-O-glucoside) in shoots originated from the apical section (SASs) of in vitro Oleaster, cultivated at density of 24 (D24). SN refers to stems of node and the numbers from 1 to 12 refer to the number of the node starting from 1 the top node; Table S7: Aromatic cytokinin conjugates (BA: benzyl-aminopurine, mT: meta-topolin, MeoT: ortho-methoxy-topolin, BA7G: benzyladenine-7-glucoside, mT9G: meta-topolin-9-glucoside, mTOG: meta-topolin-O-glucoside, pTOG: para-topolin-O-glucoside, mTROG: meta-topolin-riboside-O-glucoside) in shoots originated from the middle section (SMSs) of in vitro Oleaster, cultivated at density of 24 (D24). LN refers to leaves of node and the numbers from 1 to 13 refer to the number of the node starting from 1 the top node; Table S8: Aromatic cytokinin conjugates (BA: benzyl-aminopurine, pT: para-topolin, mT: meta-topolin, MeoT: ortho-methoxy-topolin, BA7G: benzyladenine-7-glucoside, mT9G: meta-topolin-9-glucoside, mTOG: meta-topolin-O-glucoside, pTOG: para-topolin-O-glucoside, mTROG: meta-topolin-riboside-O-glucoside) in shoots originated from the middle section (SMSs) of in vitro Oleaster, cultivated at density of 24 (D24). SN refers to stems of node and the numbers from 1 to 13 refer to the number of the node starting from 1 the top node; Table S9: Aromatic cytokinin conjugates (BA: benzyl-aminopurine, pT: para-topolin, mT: meta-topolin, MeoT: ortho-methoxy-topolin, BA7G: benzyladenine-7-glucoside, mTOG: meta-topolin-O-glucoside, pTOG: para-topolin-O-glucoside, mTROG: meta-topolin-riboside-O-glucoside) in shoots originated from the apical section (SASs) of in vitro Oleaster, cultivated at density of 30 (D30). LN refers to leaves of node and the numbers from 1 to 11 refer to the number of the node starting from 1 the top node; Table S10: Aromatic cytokinin conjugates (BA: benzyl-aminopurine, pT: para-topolin, mT: meta-topolin, MeoT: ortho-methoxy-topolin, BA7G: benzyladenine-7-glucoside, mT9G: meta-topolin-9-glucoside, mTOG: meta-topolin-O-glucoside, pTOG: para-topolin-O-glucoside, mTROG: meta-topolin-riboside-O-glucoside) in shoots originated from the apical section (SASs) of in vitro Oleaster, cultivated at density of 30 (D30). SN refers to stems of node and the numbers from 1 to 14 refer to the number of the node starting from 1 the top node; Table S11: Aromatic cytokinin conjugates (BA: benzyl-aminopurine, mT: meta-topolin, MeoT: ortho-methoxy-topolin, BA7G: benzyladenine-7-glucoside, mT9G: meta-topolin-9-glucoside, mTOG: meta-topolin-O-glucoside, pTOG: para-topolin-O-glucoside, mTROG: meta-topolin-riboside-O-glucoside) in shoots originated from the middle section (SMSs) of in vitro Oleaster, cultivated at density of 30 (D30). LN refers to leaves of node and the numbers from 1 to 13 refer to the number of the node starting from 1 the top node; Table S12: Aromatic cytokinin conjugates (BA: benzyl-aminopurine, mT: meta-topolin, MeoT: ortho-methoxy-topolin, BA7G: benzyladenine-7-glucoside, mT9G: meta-topolin-9-glucoside, mTOG: meta-topolin-O-glucoside, pTOG: para-topolin-O-glucoside, mTROG: meta-topolin-riboside-O-glucoside) in shoots originated from the middle section (SMSs) of in vitro Oleaster, cultivated at density of 30 (D30). SN refers to stems of node and the numbers from 1 to 13 refer to the number of the node starting from 1 the top node; Table S13: Isoprenoid cytokinin conjugates (tZR: trans-zeatin Riboside, tZ: trans-zeatin, iPA: isopentenyladenosine, DHZN7G: dihydrozeatin 7-glucoside, ZN7G: zeatin 7-glucoside, DHZN9G: dihydrozeatin 9-glucoside, ZN9G: zeatin 9-glucoside, DHZOG: dihydrozeatin O-glucoside, ZOG: zeatin O-glucoside, iP7G: isopentenyladenosine 7-glucoside and iP9G: isopentenyladenosine 9-glucoside) in shoots originated from the apical section (SASs) of in vitro Oleaster, cultivated at density of 18 (D18). LN refers to leaves of node and the numbers from 1 to 11 refer to the number of the node starting from 1 the top node; Table S14. Isoprenoid cytokinin conjugates (DHZR: dihydrozeatin riboside, tZR: trans-zeatin Riboside, tZ: trans-zeatin, iPA: isopentenyladenosine, DHZN7G: dihydrozeatin 7-glucoside, ZN7G: zeatin 7-glucoside, DHZN9G: dihydrozeatin 9-glucoside, ZN9G: zeatin 9-glucoside, ZOG: zeatin O-glucoside, iP7G: isopentenyladenosine 7-glucoside and iP9G: isopentenyladenosine 9-glucoside) in shoots originated from the apical section (SASs) of in vitro Oleaster, cultivated at density of 18 (D18). SN refers to stems of node and the numbers from 1 to 11 refer to the number of the node starting from 1 the top node; Table S15: Isoprenoid cytokinin conjugates (DHZR: dihydrozeatin riboside, tZR: trans-zeatin Riboside, tZ: trans-zeatin, iPA: isopentenyladenosine, DHZN7G: dihydrozeatin 7-glucoside, ZN7G: zeatin 7-glucoside, DHZN9G: dihydrozeatin 9-glucoside, ZN9G: zeatin 9-glucoside, DHZOG: dihydrozeatin O-glucoside, ZOG: zeatin O-glucoside, iP7G: isopentenyladenosine 7-glucoside and iP9G: isopentenyladenosine 9-glucoside) in shoots originated from the middle section (SMSs) of in vitro Oleaster, cultivated at density of 18 (D18). LN refers to leaves of node and the numbers from 1 to 9 refer to the number of the node starting from 1 the top node; Table S16: Isoprenoid cytokinin conjugates (DHZR: dihydrozeatin riboside, tZR: trans-zeatin Riboside, tZ: trans-zeatin, iPA: isopentenyladenosine, DHZN7G: dihydrozeatin 7-glucoside, ZN7G: zeatin 7-glucoside, DHZN9G: dihydrozeatin 9-glucoside, ZN9G: zeatin 9-glucoside, DHZOG: dihydrozeatin O-glucoside, ZOG: zeatin O-glucoside, iP7G: isopentenyladenosine 7-glucoside and iP9G: isopentenyladenosine 9-glucoside) in shoots originated from the middle section (SMSs) of in vitro Oleaster, cultivated at density of 18 (D18). SN refers to stems of node and the numbers from 1 to 9 refer to the number of the node starting from 1 the top node; Table S17: Isoprenoid cytokinin conjugates (iPA: isopentenyladenosine, DHZN7G: dihydrozeatin 7-glucoside, ZN7G: zeatin 7-glucoside, DHZN9G: dihydrozeatin 9-glucoside, ZN9G: zeatin 9-glucoside, DHZOG: dihydrozeatin O-glucoside, ZOG: zeatin O-glucoside, iP7G: isopentenyladenosine 7-glucoside and iP9G: isopentenyladenosine 9-glucoside) in shoots originated from the apical section (SAS) cultivated at density of 24 (D24). LN refers to leaves of node and the numbers from 1 to 11 refer to the number of the node starting from 1 the top node; Table S18: Isoprenoid cytokinin conjugates (DHZR: dihydrozeatin riboside, tZR: trans-zeatin Riboside, tZ: trans-zeatin, iPA: isopentenyladenosine, DHZN7G: dihydrozeatin 7-glucoside, ZN7G: zeatin 7-glucoside, DHZN9G: dihydrozeatin 9-glucoside, ZN9G: zeatin 9-glucoside, ZOG: zeatin O-glucoside, iP7G: isopentenyladenosine 7-glucoside and iP9G: isopentenyladenosine 9-glucoside) in shoots originated from the apical section (SAS) cultivated at density of 24 (D24). SN refers to stems of node and the numbers from 1 to 12 refer to the number of the node starting from 1 the top node; Table S19: Isoprenoid cytokinin conjugates (iPA: isopentenyladenosine, DHZN7G: dihydrozeatin 7-glucoside, ZN7G: zeatin 7-glucoside, DHZN9G: dihydrozeatin 9-glucoside, ZN9G: zeatin 9-glucoside, DHZOG: dihydrozeatin O-glucoside, ZOG: zeatin O-glucoside, iP7G: isopentenyladenosine 7-glucoside and iP9G: isopentenyladenosine 9-glucoside) in shoots originated from the middle section (SMS) cultivated at density of 24 (D24). LN refers to leaves of node and the numbers from 1 to 13 refer to the number of the node starting from 1 the top node; Table S20: Isoprenoid cytokinin conjugates (DHZR: dihydrozeatin riboside, tZR: trans-zeatin Riboside, iPA: isopentenyladenosine, DHZN7G: dihydrozeatin 7-glucoside, ZN9G: zeatin 9-glucoside, DHZOG: dihydrozeatin O-glucoside, ZOG: zeatin O-glucoside, iP7G: isopentenyladenosine 7-glucoside and iP9G: isopentenyladenosine 9-glucoside) in shoots originated from the middle section (SMS) cultivated at density of 24 (D24). LN refers to leaves of node and the numbers from 1 to 13 refer to the number of the node starting from 1 the top node; Table S21: Isoprenoid cytokinin conjugates (tZR: trans-zeatin Riboside, iPA: isopentenyladenosine, ZN7G: zeatin 7-glucoside DHZN9G: dihydrozeatin 9-glucoside, ZN9G: zeatin 9-glucoside, DHZOG: dihydrozeatin O-glucoside, ZOG: zeatin O-glucoside, iP7G: isopentenyladenosine 7-glucoside and iP9G: isopentenyladenosine 9-glucoside) in shoots originated from the apical section (SAS) cultivated at density of 30 (D30). LN refers to leaves of node and the numbers from 1 to 11 refer to the number of the node starting from 1 the top node; Table S22: Isoprenoid cytokinin conjugates (tZR: trans-zeatin Riboside, tZ: trans-zeatin, iPA: isopentenyladenosine, DHZN7G: dihydrozeatin 7-glucoside, ZN7G: zeatin 7-glucoside DHZN9G: dihydrozeatin 9-glucoside, ZN9G: zeatin 9-glucoside, ZOG: zeatin O-glucoside, iP7G: isopentenyladenosine 7-glucoside and iP9G: isopentenyladenosine 9-glucoside and ZROG: zeatin riboside O-glucoside) in shoots originated from the apical section (SAS) cultivated at density of 30 (D30). SN refers to stems of node and the numbers from 1 to 13 refer to the number of the node starting from 1 the top node; Table S23: Isoprenoid cytokinin conjugates (DHZR: dihydrozeatin riboside, iPA: isopentenyladenosine, ZN7G: zeatin 7-glucoside, DHZN9G: dihydrozeatin 9-glucoside, ZN9G: zeatin 9-glucoside, DHZOG: dihydrozeatin O-glucoside, ZOG: zeatin O-glucoside, iP7G: isopentenyladenosine 7-glucoside and iP9G: isopentenyladenosine 9-glucoside and ZROG: zeatin riboside O-glucoside) in shoots originated from the middle section (SMS) cultivated at density of 30 (D30). LN refers to leaves of node and the numbers from 1 to 13 refer to the number of the node starting from 1 the top node; Table S24: Isoprenoid cytokinin conjugates (tZR: trans-zeatin Riboside, iPA: isopentenyladenosine, DHZN7G: dihydrozeatin 7-glucoside, ZN7G: zeatin 7-glucoside, DHZN9G: dihydrozeatin 9-glucoside, ZN9G: zeatin 9-glucoside, DHZOG: dihydrozeatin O-glucoside, ZOG: zeatin O-glucoside, iP7G: isopentenyladenosine 7-glucoside, iP9G: isopentenyladenosine 9-glucoside, ZROG: zeatin riboside O-glucoside and DHZROG: dihydrozeatin riboside O-glucoside) in shoots originated from the middle section (SMS) cultivated at density of 30 (D30). SN refers to stems of node and the numbers from 1 to 13 refer to the number of the node starting from 1 the top node.
