# Supplementary material for: Exploring the Interplay of Explant Origin and Culture Density on Olive Micropropagation Efficiency
Source: Plants (Basel). 2025 Apr 9;14(8):1170. doi: 10.3390/plants14081170 (PMC12030091; doi:10.3390/plants14081170)
Supplement: Supplementary file 1 [file plants-14-01170-s001.zip › plants-3477997-supplementary.pdf]

Supplementary materials

Table S1. Aromatic cytokinin conjugates (BA: benzyl-aminopurine, mT: meta-topolin, MeoT: ortho-methoxy-topolin, mTOG: meta-topolin-O-glucoside, mT7G: meta-topolin-7-glucoside, pTOG: para-topolin-O-glucoside, mTROG: meta-topolin-riboside-O-glucoside) in shoots originated from the apical section (SASs) of in vitro Oleaster, cultivated at density of 18 (D18). LN refers to leaves of node and the numbers from 1 to 11 refer to the number of the node starting from 1 the top node.

	**BA**	**mT**	**MeoT**	**mTOG**	**mT7G**	**pTOG**	**mTROG**	**Aromatic free bases**	**Aromatic O-glucosides**	**Aromatic N-glucosides**
**LN1**	67±18e	ND	51±4d	1854±51a	ND	4340±53a	51±2e	119±21de	6245±102a	ND
**LN2**	120±20cde	ND	37±8d	331±20h	ND	1651±50f	156±5b	157±28de	2137±75f	ND
**LN3**	145±25c	ND	135±7c	425±25g	2±1e	1671±26f	63±3c	280±32c	2159±53f	2±1e
**LN4**	129±29cd	ND	63±7d	686±15e	17±2b	1851±50e	56±1d	192±36d	2592±66e	17±2b
**LN5**	206±25b	216±5b	64±6d	1026±25b	20±2a	1624±25f	33±3g	486±36b	2683±53e	20±2a
**LN6**	82±12de	ND	ND	981±20c	ND	2351±50c	42±2f	82±12e	3374±72c	ND
**LN7**	149±40c	289±10a	209±28b	845±25d	5±1d	2049±50d	26±1h	647±58a	2920±76d	5±1d
**LN8**	88±13de	ND	230±29b	556±15f	4±1d	1524±25g	8±1i	318±41c	2087±41f	4±1d
**LN9**	90±20de	ND	ND	550±30f	ND	1547±50g	ND	90±20e	2097±80f	ND
**LN10**	166±45bc	65±5c	387±27a	ND	16±1c	1497±50g	185±5a	617±77a	1682±55g	16±1c
**LN11**	299±50a	ND	116±16c	695±25e	ND	3451±50b	ND	414±66b	4146±75b	ND

Means followed by the Same letter are not significantly different ($p \leq 0.05$; Kruskal–Wallis test). Statistical analysis were performed for each column varying the node position (vertically per column) and letters were attributed according to the significance of the node position. L: leaves, N: node and the number indicates the node number starting from the top (1).

Table S2. Aromatic cytokinin conjugates (BA: benzyl-aminopurine, MeoT: ortho-methoxy-topolin, mTOG: meta-topolin-O-glucoside, mT7G: meta-topolin-7-glucoside, pTOG: para-topolin-O-glucoside, mTROG: meta-topolin-riboside-O-glucoside) in shoots originated from the apical section (SASs) of in vitro Oleaster, cultivated at density of 18 (D18). SN refers to stems of node and the numbers from 1 to 11 refer to the number of the node starting from 1 the top node.

	**BA**	**MeoT**	**mTOG**	**mT7G**	**pTOG**	**mTROG**	**Aromatic free bases**	**Aromatic O-glucosides**	**Aromatic N-glucosides**
**SN1**	294±14b	ND	1516±35a	ND	1375±25c	142±3d	294±14e	3033±63a	ND
**SN2**	292±12b	176±4a	ND	1±0d	1879±26a	235±5b	468±16b	2114±31b	1±0d
**SN3**	291±20b	118±3c	ND	10±2b	1624±25b	325±5a	408±23c	1948±30c	10±2b
**SN4**	415±35a	163±10b	ND	6±1c	1380±26c	98±3g	578±45a	1478±29d	6±1c
**SN5**	236±25c	85±5f	ND	17±1a	691±10d	328±8a	321±30de	1018±18g	17±1a

**SN6**	165±25d	102±7de	362±13c	ND	607±8e	122±3f	267±32ef	1091±23f	ND
**SN7**	250±50bc	111±8cd	173±13e	ND	417±8g	80±5h	361±58cd	670±25i	ND
**SN8**	110±15e	114±6c	300±20d	ND	292±10h	220±5c	224±21fg	811±35h	ND
**SN9**	88±13e	99±10e	656±15b	ND	458±8f	82±3h	187±23g	1196±25e	ND
**SN10**	256±35bc	63±3g	670±20b	ND	411±10g	133±3e	319±38de	1214±33e	ND
**SN11**	285±45bc	81±7f	100±10f	ND	170±10i	ND	366±52cd	271±20j	ND

Means followed by the Same letter are not significantly different (p ≤ 0.05; Kruskal–Wallis test). Statistical analysis were performed for each column varying the node position (vertically per column) and letters were attributed according to the significance of the node position. S: stem, N: node and the number indicates the node number starting from the top (1).

Table S3. Aromatic cytokinin conjugates (BA: benzyl-aminopurine, mT: meta-topolin, MeoT: ortho-methoxy-topolin, mTOG: meta-topolin-O-glucoside, pTOG: para-topolin-O-glucoside, mTROG: meta-topolin-riboside-O-glucoside) in shoots originated from the middle section (SMSs) of in vitro Oleaster, cultivated at density of 18 (D18). LN refers to leaves of node and the numbers from 1 to 9 refer to the number of the node starting from 1 the top node.

Aromatic cytokinin conjugates in leaves of shoots derived from the middle section (SMSs) of in vitro Oleaster, cultivated at D18.

	**BA**	**mT**	**MeoT**	**mTOG**	**pTOG**	**mTROG**	**Aromatic free bases**	**Aromatic O-glucosides**
**LN1**	75±5d	250±10d	90±30d	513±13h	2025±25i	ND	415±45d	2538±38i
**LN2**	70±5d	310±30c	96±19d	766±15g	2251±50h	ND	476±54cd	3017±65h
**LN3**	68±8d	194±15e	174±46cd	880±30f	2362±54g	40±1b	437±68d	3282±83g
**LN4**	80±10d	226±25de	170±32cd	1176±25e	2960±53f	ND	475±67cd	4136±77f
**LN5**	210±20b	273±25cd	134±36d	1154±51e	3463±55e	24±1c	617±81cd	4641±105e
**LN6**	65±15d	401±50b	1292±192a	2879±75c	3956±51d	ND	1758±257a	6835±126d
**LN7**	375±25a	ND	372±74c	2101±100d	5248±50b	172±3a	747±99c	7520±153c
**LN8**	160±20c	ND	1128±229a	3276±75a	5358±52a	ND	1288±249b	8635±126a
**LN9**	360±30a	586±56a	857±157b	3051±100b	4923±25c	ND	1803±238a	7974±125b

Means followed by the Same letter are not significantly different (p ≤ 0.05; Kruskal–Wallis test). Statistical analysis were performed for each column varying the node position (vertically per column) and letters were attributed according to the significance of the node position. L: leaves, N: node and the number indicates the node number starting from the top (1).

Table S4. Aromatic cytokinin conjugates (BA: benzyl-aminopurine, MeoT: ortho-methoxy-topolin, mT9G: meta-topolin-9-glucoside, mTOG: meta-topolin-O-glucoside, pTOG: para-topolin-O-glucoside, mTROG: meta-topolin-riboside-O-glucoside, MemT9G: meta-methoxy-topolin-9-glucoside) in shoots originated from the middle section (SMSs) of in vitro Oleaster, cultivated at density of 18 (D18). SN refers to stems of node and the numbers from 1 to 9 refer to the number of the node starting from 1 the top node.

	BA	MeoT	mT9G	mTOG	pTOG	mTROG	MemT9G	Aromatic free bases	Aromatic O-glucosides	Aromatic N-glucosides
**SN1**	553±50a	55±5h	ND	470±20i	1705±5a	175±5b	ND	608±55c	2350±30e	ND
**SN2**	390±20b	248±18f	498±20d	960±20h	1005±5d	ND	ND	638±38c	1966±25g	498±20e
**SN3**	220±20de	716±26a	547±50d	1116±15g	490±10g	55±1e	267±28a	936±46a	1661±26i	814±78d
**SN4**	320±20c	525±20c	492±30d	2022±26d	805±5e	225±5a	ND	845±40b	3052±35d	492±30e
**SN5**	115±15f	194±15g	565±35d	1512±13e	256±6h	ND	203±62b	308±30d	1768±18h	768±27d
**SN6**	331±30c	604±25b	2242±151a	4646±50a	992±11d	58±3e	117±16c	935±55a	5697±63a	2359±166a
**SN7**	185±15e	461±24d	1344±120c	2803±50c	1197±15b	106±5c	ND	646±39c	4106±71c	1344±150c
**SN8**	248±18d	530±30c	1553±150b	3405±51b	1052±11c	ND	ND	778±47b	4457±61b	1553±150b
**SN9**	220±20de	402±23e	571±75d	1416±15f	656±15f	71±1d	ND	622±43c	2143±31f	571±75e

Means followed by the Same letter are not significantly different ($p \leq 0.05$; Kruskal–Wallis test). Statistical analysis were performed for each column varying the node position (vertically per column) and letters were attributed according to the significance of the node position. S: stem, N: node and the number indicates the node number starting from the top (1).

Table S5. Aromatic cytokinin conjugates (BA: benzyl-aminopurine, pT: para-topolin, mT: meta-topolin, MeoT: ortho-methoxy-topolin, mTOG: meta-topolin-O-glucoside, pTOG: para-topolin-O-glucoside) in shoots originated from the apical section (SASs) of in vitro Oleaster, cultivated at density of 24 (D24). LN refers to leaves of node and the numbers from 1 to 11 refer to the number of the node starting from 1 the top node.

	BA	pT	mT	MeoT	mTOG	pTOG	mTROG	Aromatic free bases	Aromatic O-glucosides
**LN1**	389±20ef	75±7b	77±8c	42±3i	1066±15a	3332±28a	97±3h	584±37e	4496±45a
**LN2**	130±10h	78±1ab	ND	315±15d	953±13b	ND	355±5b	523±26e	1308±18d
**LN3**	230±20g	65±5c	75±15c	153±13g	ND	ND	284±5c	523±53e	284±5i
**LN4**	ND	43±8d	ND	533±21a	ND	2774±25b	212±3e	575±28e	2986±28b
**LN5**	625±25c	83±6a	ND	208±15f	ND	ND	195±5f	916±45c	195±5j
**LN6**	441±20e	ND	ND	91±10h	793±13c	ND	285±5c	532±30e	1077±18f
**LN7**	376±25f	ND	145±15a	266±17e	ND	1566±15c	ND	787±57d	1566±15c
**LN8**	665±25bc	ND	95±15b	412±13c	ND	906±5d	366±5a	1172±53b	1271±10e
**LN9**	1277±75a	ND	ND	ND	ND	ND	172±3g	1277±75a	172±3j
**LN10**	712±30b	ND	ND	465±16b	ND	406±5e	256±5d	1178±46b	662±10h
**LN11**	531±30d	ND	ND	430±30c	676±25d	ND	73±3i	961±60c	749±28g

Means followed by the Same letter are not significantly different ($p \leq 0.05$; Kruskal–Wallis test). Statistical analysis were performed for each column varying the node position (vertically per column) and letters were attributed according to the significance of the node position. L: leaves, N: node and the number indicates the node number starting from the top (1).

Table S6. Aromatic cytokinin conjugates (BA: benzyl-aminopurine, pT: para-topolin, mT: meta-topolin MeoT: ortho-methoxy-topolin, BA7G: benzyladenine-7-glucoside, mT9G: meta-topolin-9-glucoside, mTOG: meta-topolin-O-glucoside, pTOG: para-topolin-O-glucoside, mTROG: meta-topolin-riboside-O-glucoside) in shoots originated from the apical section (SASs) of in vitro Oleaster, cultivated at density of 24 (D24). SN refers to stems of node and the numbers from 1 to 12 refer to the number of the node starting from 1 the top node.

	**BA**	**pT**	**mT**	**MeoT**	**BA7G**	**mT9G**	**mTOG**	**pTOG**	**mTROG**	**Aromatic free bases**	**Aromatic O-glucosides**	**Aromatic N-glucosides**
**SN1**	ND	165±15a	ND	328±8c	ND	ND	2201±50b	3075±25b	640±10h	493±23d	5916±85b	ND
**SN2**	262±13d	46±6b	30±5b	330±10c	ND	ND	1253±50f	3201±20a	1070±10c	668±34c	5524±80c	ND
**SN3**	160±10g	20±5c	ND	157±8e	ND	ND	ND	2612±13c	676±5g	338±23e	3287±18g	ND
**SN4**	170±10g	ND	ND	198±8d	ND	329±17d	496±15h	1675±25g	435±5k	368±18e	2606±45h	329±17e
**SN5**	195±15fg	ND	35±5a	140±10e	ND	202±17e	350±10i	2126±25e	968±16d	370±30e	3444±51f	202±17f
**SN6**	75±15h	ND	ND	120±3f	ND	691±29c	1723±25d	1927±25f	506±5j	195±18f	4156±55d	691±29c
**SN7**	270±20d	ND	ND	116±5f	ND	1166±113b	3704±51a	1200±20h	585±5i	386±25e	5490±75c	1166±113b
**SN8**	256±15de	ND	ND	595±15b	ND	1240±62a	1979±26c	1166±15h	730±10f	850±30b	3875±51e	1240±62a
**SN9**	326±25c	ND	25±5c	ND	ND	ND	341±10i	636±15i	791±10e	350±30e	1768±35j	ND
**SN10**	686±35b	ND	ND	ND	ND	ND	450±10h	605±15i	1255±5b	686±35c	2310±30i	ND
**SN11**	215±15ef	ND	33±3ab	151±10e	124±11	287±32d	1461±20e	250±10j	606±5i	398±28e	2317±35i	411±21d
**SN12**	1562±60a	ND	ND	905±25a	ND	ND	1188±35g	2499±50d	6552±50a	2466±85a	10239±135a	ND

Means followed by the Same letter are not significantly different ($p \leq 0.05$; Kruskal–Wallis test). Statistical analysis were performed for each column varying the node position (vertically per column) and letters were attributed according to the significance of the node position. S: stem, N: node and the number indicates the node number starting from the top (1).

Table S7. Aromatic cytokinin conjugates (BA: benzyl-aminopurine, mT: meta-topolin, MeoT: ortho-methoxy-topolin, BA7G: benzyladenine-7-glucoside, mT9G: meta-topolin-9-glucoside, mTOG: meta-topolin-O-glucoside, pTOG: para-topolin-O-glucoside, mTROG: meta-topolin-riboside-O-glucoside) in shoots originated from the middle section (SMSs) of in vitro Oleaster, cultivated at density of 24 (D24). LN refers to leaves of node and the numbers from 1 to 13 refer to the number of the node starting from 1 the top node.

	BA	mT	MeoT	BA7G	mT9G	mTOG	pTOG	mTROG	Aromatic free bases	Aromatic O-glucosides	Aromatic N-glucosides
**LN1**	32±8f	ND	211±12d	48±6e	150±47c	1291±10b	2811±10a	52±3hi	244±20hi	4153±23b	198±41bcd
**LN2**	225±15c	32±3g	328±13c	65±3cd	ND	827±15e	2829±26a	88±3d	585±30d	3743±44c	65±3ef
**LN3**	130±10d	ND	330±20c	71±5c	ND	876±15d	2377±25d	135±5e	470±30e	3389±45d	71±5ef
**LN4**	135±15d	45±5g	154±13e	53±5de	ND	766±15f	2010±10g	85±5f	334±33f	2861±30h	53±5f
**LN5**	48±8f	75±5f	196±15d	65±6cd	ND	810±10e	2192±11f	103±3fg	319±28fg	3105±23g	65±6ef
**LN6**	85±5e	12±3h	140±15e	89±7b	ND	701±10g	2525±25b	68±3i	237±18i	3294±38e	89±7def
**LN7**	52±8ff	82±2ef	122±13e	93±8b	ND	491±10i	1879±26h	82±3ghi	256±22ghi	2452±38j	93±8def
**LN8**	105±10e	92±3e	115±15e	61±4cde	ND	366±15j	1592±10j	156±5fgh	313±28efg	2113±30k	61±4ef
**LN9**	100±10e	135±5de	588±35b	60±6cde	151±49c	931±10c	1554±6k	83±3c	824±50c	2568±18i	211±42bc
**LN10**	140±10d	265±15c	ND	ND	296±37b	810±10e	2232±28e	228±8e	405±25e	3270±44ef	296±37b
**LN11**	243±13c	ND	ND	ND	ND	655±15h	2415±15c	148±8hi	243±13hi	3219±38f	ND
**LN12**	450±20b	440±20b	151±20e	ND	1441±211a	2576±25a	1777±25i	666±15b	1041±60b	5019±65a	1441±211a
**LN13**	595±25a	481±20a	901±50a	128±27a	ND	ND	1464±15l	188±8a	1978±95a	1652±23l	128±27cde

Means followed by the Same letter are not significantly different (p ≤ 0.05; Kruskal–Wallis test). Statistical analysis were performed for each column varying the node position (vertically per column) and letters were attributed according to the significance of the node position. L: leaves, N: node and the number indicates the node number starting from the top (1).

Table S8. Aromatic cytokinin conjugates (BA: benzyl-aminopurine, pT: para-topolin, mT: meta-topolin, MeoT: ortho-methoxy-topolin, BA7G: benzyladenine-7-glucoside, mT9G: meta-topolin-9-glucoside, mTOG: meta-topolin-O-glucoside, pTOG: para-topolin-O-glucoside, mTROG: meta-topolin-riboside-O-glucoside) in shoots originated from the middle section (SMSs) of in vitro Oleaster, cultivated at density of 24 (D24). SN refers to stems of node and the numbers from 1 to 13 refer to the number of the node starting from 1 the top node.

	BA	pT	mT	MeoT	BA7G	mT9G	mTOG	pTOG	mTROG	Aromatic free bases	Aromatic O-glucosides	Aromatic N-glucosides
**SN1**	440±20c	52±4a	ND	108±8g	18±3i	ND	920±20d	1577±25b	286±5d	600±32c	2783±50c	18±3f
**SN2**	665±15a	11±3b	ND	102±8g	111±3de	150±10e	655±15f	1607±11a	595±5b	779±26b	2857±31c	261±7e
**SN3**	76±5h	13±3b	28±3c	35±5h	97±3f	478±25d	776±15e	1410±10d	316±5c	151±15g	2502±30e	574±22d
**SN4**	64±4h	ND	8±3e	111±10g	94±4f	426±25d	755±15e	1124±25g	83±3i	182±16g	1962±43f	520±22d
**SN5**	166±15f	ND	ND	ND	76±4g	ND	141±10i	1132±28g	262±3e	166±15g	1535±40g	76±4ef
**SN6**	80±5h	ND	ND	153±8f	93±3f	181±20e	466±15g	1293±11e	252±3f	233±13f	2011±28f	273±18e
**SN7**	121±10g	ND	ND	148±8f	118±3cd	ND	ND	1229±26f	122±3g	268±18f	1351±28h	118±3ef
**SN8**	466±15b	ND	32±3b	285±15d	143±3b	ND	420±20g	1466±15c	714±13a	783±33b	2601±48d	143±3ef
**SN9**	330±20d	ND	ND	ND	169±8a	ND	ND	935±15h	95±5h	330±20e	1030±20i	169±8ef

**SN10**	331±30d	ND	23±3de	220±20e	120±5c	127±25e	343±13h	444±5k	73±3j	574±53cd	860±20j	247±21e
**SN11**	110±10g	ND	37±3a	384±15c	116±4cde	1459±151c	1402±50c	511±10j	47±3k	531±28d	1960±63f	1575±147c
**SN12**	435±15c	ND	ND	445±15b	109±7e	2407±200b	5275±75a	610±10i	90±5hi	880±30a	5976±90a	2516±194b
**SN13**	240±10e	ND	27±3c	482±13a	67±5h	6096±300a	4980±75b	350±10l	46±2k	749±25b	5376±87b	6163±295a

Means followed by the Same letter are not significantly different ($p \leq 0.05$; Kruskal–Wallis test). Statistical analysis were performed for each column varying the node position (vertically per column) and letters were attributed according to the significance of the node position. S: stem, N: node and the number indicates the node number starting from the top (1).

Table S9. Aromatic cytokinin conjugates (BA: benzyl-aminopurine, pT: para-topolin, mT: meta-topolin, MeoT: ortho-methoxy-topolin, BA7G: benzyladenine-7-glucoside, mTOG: meta-topolin-O-glucoside, pTOG: para-topolin-O-glucoside, mTROG: meta-topolin-riboside-O-glucoside) in shoots originated from the apical section (SASs) of in vitro Oleaster, cultivated at density of 30 (D30). LN refers to leaves of node and the numbers from 1 to 11 refer to the number of the node starting from 1 the top node.

Aromatic cytokinin conjugates in leaves of shoots derived from the apical section (SASs) of in vitro Oleaster, cultivated at D30.

	**BA**	**pT**	**mT**	**MeoT**	**BA7G**	**mTOG**	**pTOG**	**mTROG**	**Aromatic free bases**	**Aromatic O-glucosides**	**Aromatic N-glucosides**
**LN1**	510±20c	ND	67±7d	128±13h	ND	960±10a	ND	113±3i	705±39f	1073±13c	ND
**LN2**	481±20c	ND	71±3	ND	ND	525±5f	ND	57±3k	552±17g	583±8h	ND
**LN3**	936±25a	ND	ND	540±20b	ND	ND	38±3c	675±5a	1476±45a	712±8g	ND
**LN4**	910±30a	ND	ND	275±25f	54±4e	ND	ND	505±5d	1185±55c	505±5i	54±4e
**LN5**	575±25b	ND	ND	355±15e	ND	102±3g	636±15b	215±5f	930±40e	953±23d	ND
**LN6**	580±30b	ND	ND	490±20c	65±3d	ND	ND	552±3c	1071±50d	552±3h	65±3d
**LN7**	235±15f	51±4a	259±50a	215±15g	73±3c	780±10c	ND	122±3h	761±84f	902±13e	73±3c
**LN8**	312±13e	33±4b	172±20c	450±20d	80±5b	580±10e	10±2d	175±5g	968±56e	765±17f	80±5b
**LN9**	390±20d	ND	224±16b	743±40a	57±3e	ND	1126±25a	88±3j	1357±76b	1214±28b	57±3e
**LN10**	248±18f	ND	ND	239±20fg	89±4a	656±15d	635±15b	586±5b	487±38g	1876±35a	89±4a
**LN11**	930±30a	53±5a	ND	155±25h	63±3d	844±15b	5±1d	226±5e	1138±60cd	1075±21c	63±3d

Means followed by the Same letter are not significantly different ($p \leq 0.05$; Kruskal–Wallis test). Statistical analysis were performed for each column varying the node position (vertically per column) and letters were attributed according to the significance of the node position. L: leaves, N: node and the number indicates the node number starting from the top (1).

Table S10. Aromatic cytokinin conjugates (BA: benzyl-aminopurine, pT: para-topolin, mT: meta-topolin, MeoT: ortho-methoxy-topolin, BA7G: benzyladenine-7-glucoside, mT9G: meta-topolin-9-glucoside, mTOG: meta-topolin-O-glucoside, pTOG: para-topolin-O-glucoside, mTROG: meta-topolin-riboside-O-glucoside) in shoots originated from

the apical section (SASs) of in vitro Oleaster, cultivated at density of 30 (D30). SN refers to stems of node and the numbers from 1 to 14 refer to the number of the node starting from 1 the top node.

	BA	pT	mT	MeoT	BA7G	mT9G	mTOG	pTOG	mTROG	oT9G	Aromatic free bases	Aromatic O-glucosides	Aromatic N-glucosides
**SN1**	851±30b	ND	50±3c	ND	ND	ND	1890±10a	ND	575±5e	ND	901±33c	2465±5e	ND
**SN2**	425±15d	ND	ND	ND	94±4d	35±5f	1601±20b	ND	2078±26b	ND	425±15f	3679±9c	129±1f
**SN3**	85±5j	ND	ND	ND	74±4fg	ND	926±25f	3267±15a	1625±25c	ND	85±5h	5818±16a	74±4h
**SN4**	965±35a	ND	ND	230±10c	133±3b	ND	270±10h	2304±5b	1387±15d	ND	1195±45b	3961±10b	133±3f
**SN5**	405±15def	ND	ND	165±15de	122±4c	ND	ND	1210±10c	2132±28a	ND	570±30e	3342±37d	122±4fg
**SN6**	425±15d	645±25a	ND	5224±125a	85±5e	ND	ND	1054±5d	550±10f	ND	6294±165a	1604±15k	85±5gh
**SN7**	385±15ef	14±4b	34±2d	111±10ef	77±4f	214±15e	652±50g	905±5e	336±5g	ND	543±30e	1893±40i	291±12e
**SN8**	373±13f	8±2b	ND	157±8de	78±4f	320±20c	1181±27d	590±10i	296±5h	ND	538±22e	2067±15h	398±16d
**SN9**	470±20c	ND	ND	205±10cd	79±4ef	485±15a	1421±26c	730±10g	335±5g	86±11c	675±30d	2486±12e	650±22b
**SN10**	286±15g	ND	175±5a	175±15cd	ND	473±25a	652±50g	8±1l	98±3j	ND	636±35de	758±47l	473±25c
**SN11**	418±18de	ND	ND	ND	155±5a	ND	1129±26d	813±11f	182±3i	ND	418±18f	2124±17g	155±5f
**SN12**	220±20h	ND	ND	374±15b	77±4f	435±15b	1052±50e	604±5h	100±5j	202±56a	595±35de	1756±40j	713±68a
**SN13**	128±8i	ND	95±3b	87±8f	69±4g	274±25d	1550±30b	540±10j	320±10g	144±25b	310±18g	2411±10f	487±4c
**SN14**	310±20g	ND	ND	325±25b	42±2h	213±35e	1549±50b	505±5k	ND	ND	635±45de	2054±45h	256±33e

Means followed by the Same letter are not significantly different ($p \leq 0.05$; Kruskal–Wallis test). Statistical analysis were performed for each column varying the node position (vertically per column) and letters were attributed according to the significance of the node position. S: stem, N: node and the number indicates the node number starting from the top (1).

Table S11. Aromatic cytokinin conjugates (BA: benzyl-aminopurine, mT: meta-topolin, MeoT: ortho-methoxy-topolin, BA7G: benzyladenine-7-glucoside, mT9G: meta-topolin-9-glucoside, mTOG: meta-topolin-O-glucoside, pTOG: para-topolin-O-glucoside, mTROG: meta-topolin-riboside-O-glucoside) in shoots originated from the middle section (SMSs) of in vitro Oleaster, cultivated at density of 30 (D30). LN refers to leaves of node and the numbers from 1 to 13 refer to the number of the node starting from 1 the top node.

	BA	mT	MeoT	BA7G	mT9G	mTOG	pTOG	mTROG	Aromatic free bases	Aromatic O-glucosides	Aromatic N-glucosides
**LN1**	110±10g	ND	58±4g	ND	ND	1666±15e	2067±15h	43±3h	169±14h	3776±6i	ND
**LN2**	340±20d	ND	109±7f	23±2g	ND	956±15j	ND	73±3e	449±27f	1029±13m	23±2h
**LN3**	236±15e	13±3f	99±9f	23±3g	ND	516±15k	1628±26j	38±3h	347±21g	2182±15l	23±3h
**LN4**	12±7h	121±11c	46±6gh	54±4e	ND	1291±10hi	3410±10a	53±3g	179±23h	4753±3d	54±4fg

**LN5**	23±8h	43±3e	29±5h	82±4b	ND	2150±50c	3126±25c	97±3c	95±15i	5374±23a	82±4e
**LN6**	355±15d	48±3e	172±8e	43±3f	ND	1311±10h	2112±11g	63±3f	575±20e	3486±6j	43±3gh
**LN7**	186±15f	ND	170±10e	152±8a	ND	1426±25g	2704±5f	52±3g	356±25g	4182±18g	152±8d
**LN8**	121±10g	50±2e	177±8e	68±5cd	ND	1493±11f	3176±25b	ND	348±19g	4669±16e	68±5ef
**LN9**	235±15e	82±2d	302±12c	60±5de	ND	1257±21i	1967±15i	88±3d	619±29de	3312±3k	60±5efg
**LN10**	541±20b	ND	104±9f	161±10a	1803±50a	3325±25a	1311±10l	188±8b	644±29d	4823±8c	1964±41a
**LN11**	466±15c	249±4a	232±13d	79±7b	340±20c	1283±21hi	2925±25d	53±3g	947±32c	4261±9f	419±14c
**LN12**	226±15e	189±4b	1056±25a	74±6bc	976±25b	2553±50b	2782±28e	ND	1470±44b	5334±30b	1050±19b
**LN13**	1110±30a	ND	505±15b	ND	ND	1712±35d	1568±16k	745±15a	1615±45a	4025±5h	ND

Means followed by the Same letter are not significantly different (p ≤ 0.05; Kruskal–Wallis test). Statistical analysis were performed for each column varying the node position (vertically per column) and letters were attributed according to the significance of the node position. L: leaves, N: node and the number indicates the node number starting from the top (1).

Table S12. Aromatic cytokinin conjugates (BA: benzyl-aminopurine, mT: meta-topolin, MeoT: ortho-methoxy-topolin, BA7G: benzyladenine-7-glucoside, mT9G: meta-topolin-9-glucoside, mTOG: meta-topolin-O-glucoside, pTOG: para-topolin-O-glucoside, mTROG: meta-topolin-riboside-O-glucoside) in shoots originated from the middle section (SMSs) of in vitro Oleaster, cultivated at density of 30 (D30). SN refers to stems of node and the numbers from 1 to 13 refer to the number of the node starting from 1 the top node.

	**BA**	**mT**	**MeoT**	**BA7G**	**mT9G**	**mTOG**	**pTOG**	**mTROG**	**Aromatic free bases**	**Aromatic O-glucosides**	**Aromatic N-glucosides**
**SN1**	295±15a	31±1c	157±8b	25±3j	ND	1851±10c	1830±26b	258±3c	484±24a	3938±20c	25±3m
**SN2**	221±10d	ND	106±6d	53±3g	ND	ND	5±0m	245±5d	327±16c	250±5l	53±3l
**SN3**	231±10bcd	ND	ND	103±3b	256±5f	590±10h	1926±25a	212±3e	231±10fg	2728±18f	359±3g
**SN4**	281±10a	23±1d	130±5c	71±3f	ND	790±10g	1773±25c	195±5f	434±16b	2758±20e	71±3k
**SN5**	176±15e	ND	112±2d	104±4b	ND	100±10j	371±10k	385±5a	288±17d	856±5k	104±4i
**SN6**	155±15f	13±1e	104±6d	91±3d	575±5c	1352±11d	1286±15d	148±3g	272±22de	2786±10d	666±2c
**SN7**	211±10d	8±1g	27±2f	105±5b	345±5e	871±10f	836±15f	118±3h	245±12ef	1824±8h	450±1f
**SN8**	226±15cd	10±1f	107±7d	133±3a	509±10d	1086±15e	755±15g	105±5i	342±22c	1946±6g	642±7d
**SN9**	83±8h	76±2a	162±8ab	43±3h	146±5g	385±15i	1140±20e	32±3m	322±17c	1558±8i	189±3h
**SN10**	251±10b	43±2b	110±5d	100±5bc	1391±10b	4330±26a	579±10h	95±5j	404±17b	5004±12a	1491±5b
**SN11**	245±15bc	ND	168±8a	83±3e	ND	411±10i	470±10j	310±10b	413±23b	1191±10j	83±3j
**SN12**	57±8i	6±1h	124±4c	94±4cd	1586±15a	4153±50b	505±15i	60±5l	187±11h	4717±30b	1681±11a
**SN13**	117±8g	ND	89±4e	34±4i	586±15c	861±10f	260±10l	85±5k	206±12gh	1206±5j	621±11e

Means followed by the Same letter are not significantly different ($p \leq 0.05$; Kruskal–Wallis test). Statistical analysis were performed for each column varying the node position (vertically per column) and letters were attributed according to the significance of the node position. S: stem, N: node and the number indicates the node number starting from the top (1).

Table S13. Isoprenoid cytokinin conjugates (tZR: trans-zeatin Riboside, tZ: trans-zeatin, iPA: isopentenyladenosine, DHZN7G: dihydrozeatin 7-glucoside, ZN7G: zeatin 7-glucoside, DHZN9G: dihydrozeatin 9-glucoside, ZN9G: zeatin 9-glucoside, DHZOG: dihydrozeatin O-glucoside, ZOG: zeatin O-glucoside, iP7G: isopentenyladenosine 7-glucoside and iP9G: isopentenyladenosine 9-glucoside) in shoots originated from the apical section (SASs) of in vitro Oleaster, cultivated at density of 18 (D18). LN refers to leaves of node and the numbers from 1 to 11 refer to the number of the node starting from 1 the top node.

	**tZR**	**tZ**	**iPA**	**DHZN7G**	**ZN7G**	**DHZN9G**	**ZN9G**	**DHZOG**	**ZOG**	**iP7G**	**iP9G**	**Isoprenoid Free bases**	**Isoprenoid ribosides**	**Isoprenoid O-glucosides**	**Isoprenoid N-glucosides**
**LN1**	ND	ND	ND	ND	2203±100i	ND	12±3f	66±5a	118±3f	31±1h	475±25h	ND	ND	183±2d	2722±72i
**LN2**	ND	ND	ND	ND	2506±101i	ND	15±5ef	ND	117±3f	21±1g	776±25g	ND	ND	117±3f	3318±70i
**LN3**	ND	ND	6±2e	ND	7355±150h	12±3c	25±5ef	61±5b	85±5g	11±1f	926±25f	6±2d	ND	147±1e	8328±122h
**LN4**	ND	ND	ND	ND	11128±352g	ND	ND	ND	ND	43±3de	1397±50de	ND	ND	ND	12569±300g
**LN5**	6±2a	ND	14±1cd	ND	15724±254f	ND	72±3d	ND	186±6d	58±3de	1449±50de	14±1c	6±2a	186±6d	17303±200f
**LN6**	ND	48±4a	18±4c	ND	24487±501d	ND	123±3b	ND	152±3e	53±3b	2098±50b	66±8a	ND	152±3e	26760±446d
**LN7**	ND	ND	18±1c	ND	31137±351c	ND	125±5b	ND	ND	72±3c	1903±50c	18±1c	ND	ND	33238±293c
**LN8**	ND	ND	28±4a	200±5a	24485±501d	ND	106±5c	ND	967±16a	63±3c	1860±53c	28±4b	ND	967±16a	26714±438d
**LN9**	ND	ND	23±2b	118±3b	35770±751b	53±3b	120±5b	ND	611±10c	106±5de	1377±25de	23±2b	ND	611±10c	37544±716b
**LN10**	ND	ND	14±4cd	ND	22803±756e	ND	ND	ND	680±10b	67±3e	1348±50e	14±4c	ND	680±10b	24218±703e
**LN11**	ND	ND	13±3d	ND	91117±2262a	190±10a	326±15a	ND	ND	311±10a	2203±100a	13±3c	ND	ND	94147±2147a

Means followed by the Same letter are not significantly different ($p \leq 0.05$; Kruskal–Wallis test). Statistical analysis were performed for each column varying the node position (vertically per column) and letters were attributed according to the significance of the node position. L: leaves, N: node and the number indicates the node number starting from the top (1).

Table S14. Isoprenoid cytokinin conjugates (DHZR : dihydrozeatin riboside, tZR: trans-zeatin Riboside, tZ: trans-zeatin, iPA: isopentenyladenosine, DHZN7G: dihydrozeatin 7-glucoside, ZN7G: zeatin 7-glucoside, DHZN9G: dihydrozeatin 9-glucoside, ZN9G: zeatin 9-glucoside, ZOG: zeatin O-glucoside, iP7G: isopentenyladenosine 7-glucoside and iP9G: isopentenyladenosine 9-glucoside) in shoots originated from the apical section (SASs) of in vitro Oleaster, cultivated at density of 18 (D18). SN refers to stems of node and the numbers from 1 to 11 refer to the number of the node starting from 1 the top node.

	DHZR	tZR	tZ	iPA	DHZN7G	ZN7G	DHZN9G	ZN9G	ZOG	iP7G	iP9G	Isoprenoid Free bases	Isoprenoid ribosides	Isoprenoid O-glucosides	Isoprenoid N-glucosides
**SN1**	ND	ND	ND	ND	ND	3255±51h	ND	27±3g	235±5h	190±5a	1649±50d	ND	ND	235±5h	5121±10j
**SN2**	ND	ND	ND	46±1b	ND	5327±111f	ND	43±3f	320±10f	170±5b	2051±50b	46±1c	ND	320±10f	7591±65f
**SN3**	ND	ND	ND	41±1c	ND	3195±100h	ND	62±3ef	ND	70±5g	2155±51a	41±1d	ND	ND	5483±43i
**SN4**	ND	ND	ND	33±2e	ND	3903±100g	ND	ND	ND	87±3f	1778±26c	33±2f	ND	ND	5768±73h
**SN5**	ND	6±2	ND	30±2f	ND	5457±151e	ND	70±5d	255±5g	82±3f	1536±32e	30±2g	6±2b	255±5g	7145±115g
**SN6**	ND	ND	ND	41±1c	ND	10837±152c	17±2f	ND	850±10d	110±5e	1266±15f	41±1d	ND	850±10d	12230±133e
**SN7**	ND	ND	ND	38±2d	73±3b	15206±200d	34±2e	73±3d	1185±5b	140±5c	1065±15g	38±2e	ND	1185±5b	16591±177c
**SN8**	ND	ND	ND	61±2a	68±3c	12877±125a	42±3d	83±3c	666±15e	117±3d	850±20i	61±2b	ND	666±15e	14037±100d
**SN9**	9±4a	ND	56±4a	31±1ef	205±5a	25365±152d	78±3b	185±5a	1831±27a	118±3d	941±36h	87±2a	9±4a	1831±27a	26891±111a
**SN10**	ND	ND	ND	48±2b	74±4b	12877±175d	94±2a	136±5b	1030±10c	125±5d	761±20j	48±2c	ND	1030±10c	14066±143d
**SN11**	ND	ND	ND	26±1g	ND	16081±175b	68±3c	85±5c	ND	65±5g	601±20k	26±1h	ND	ND	16899±148b

Means followed by the Same letter are not significantly different (p ≤ 0.05; Kruskal–Wallis test). Statistical analysis were performed for each column varying the node position (vertically per column) and letters were attributed according to the significance of the node position. S: stem, N: node and the number indicates the node number starting from the top (1).

Table S15. Isoprenoid cytokinin conjugates (DHZR : dihydrozeatin riboside, tZR: trans-zeatin Riboside, tZ: trans-zeatin, iPA: isopentenyladenosine, DHZN7G: dihydrozeatin 7-glucoside, ZN7G: zeatin 7-glucoside, DHZN9G: dihydrozeatin 9-glucoside, ZN9G: zeatin 9-glucoside, DHZOG: dihydrozeatin O-glucoside, ZOG: zeatin O-glucoside, iP7G: isopentenyladenosine 7-glucoside and iP9G: isopentenyladenosine 9-glucoside) in shoots originated from the middle section (SMSs) of in vitro Oleaster, cultivated at density of 18 (D18). LN refers to leaves of node and the numbers from 1 to 9 refer to the number of the node starting from 1 the top node.

	DHZR	tZR	tZ	iPA	ZN7G	DHZN9G	ZN9G	DHZOG	ZOG	iP7G	iP9G	Isoprenoid Free bases	Isoprenoid ribosides	Isoprenoid O-glucosides	Isoprenoid N-glucosides
**LN1**	ND	ND	66±6b	11±1bc	1750±50	36±1	22±2	118±11	ND	37±2	759±52	77±7	ND	118±11	2604±16
**LN2**	ND	ND	ND	12±1b	2658±52	36±1	ND	135±8	ND	43±3	1104±100	12±1	ND	135±8	3840±56
**LN3**	ND	ND	ND	ND	6445±51	ND	ND	126±4	ND	24±2	1226±75	ND	ND	126±4	7695±28
**LN4**	ND	ND	ND	ND	9245±150	23±3	ND	158±9	ND	37±3	1470±75	ND	ND	158±9	10775±77
**LN5**	ND	ND	ND	ND	10339±151	74±2	ND	ND	213±3	110±5	1701±100	ND	ND	213±3	12223±50
**LN6**	101±10a	78±7a	292±11a	27±2a	19035±152	236±5	208±8	ND	ND	98±3	1411±102	319±13	179±17	ND	20987±45
**LN7**	ND	ND	ND	ND	10713±258	ND	ND	ND	ND	63±3	908±101	ND	ND	ND	11684±156
**LN8**	ND	ND	ND	10±1c	17244±250	ND	ND	291±12	ND	51±3	769±154	10±1	ND	291±12	18064±100
**LN9**	ND	ND	ND	ND	33230±252	ND	ND	ND	ND	ND	1946±150	ND	ND	ND	35177±108

Means followed by the Same letter are not significantly different (p ≤ 0.05; Kruskal–Wallis test). Statistical analysis were performed for each column varying the node position (vertically per column) and letters were attributed according to the significance of the node position. S: stem, N: node and the number indicates the node number starting from the top (1).

Table S16. Isoprenoid cytokinin conjugates (DHZR : dihydrozeatin riboside, tZR: trans-zeatin Riboside, tZ: trans-zeatin, iPA: isopentenyladenosine, DHZN7G: dihydrozeatin 7-glucoside, ZN7G: zeatin 7-glucoside, DHZN9G: dihydrozeatin 9-glucoside, ZN9G: zeatin 9-glucoside, DHZOG: dihydrozeatin O-glucoside, ZOG: zeatin O-glucoside, iP7G: isopentenyladenosine 7-glucoside and iP9G: isopentenyladenosine 9-glucoside) in shoots originated from the middle section (SMSs) of in vitro Oleaster, cultivated at density of 18 (D18). SN refers to stems of node and the numbers from 1 to 9 refer to the number of the node starting from 1 the top node.

	**DHZR**	**tZR**	**tZ**	**iPA**	**DHZN7G**	**ZN7G**	**DHZN9G**	**ZN9G**	**ZOG**	**iP7G**	**iP9G**	**Isoprenoid Free bases**	**Isoprenoid ribosides**	**Isoprenoid O-glucosides**
**SN1**	ND	ND	ND	26±1d	147±8d	17606±100h	84±2c	ND	ND	195±5b	1098±50ab	26±1h	ND	ND
**SN2**	ND	17±3c	ND	18±2f	258±8b	23607±101g	173±3b	236±5b	ND	177±3b	1052±50ab	18±2i	17±3d	ND
**SN3**	110±10a	40±5a	145±5	13±3g	263±13b	25080±76f	104±5bc	ND	ND	75±5b	822±75bc	158±8f	150±15a	ND
**SN4**	ND	ND	343±3	33±2c	ND	33408±101d	ND	ND	ND	165±5b	1175±75a	376±5c	ND	ND
**SN5**	ND	ND	ND	67±3a	232±13c	27212±102e	ND	ND	1790±10a	240±5ab	654±51c	67±3g	ND	1790±10a
**SN6**	ND	ND	438±3	29±2d	516±15a	79005±200a	ND	238±18b	961±10c	178±3b	1298±50a	467±5a	ND	961±10c
**SN7**	ND	ND	330±5	22±2e	ND	38679±75c	99±1c	ND	1568±16b	168±3b	1376±75a	352±7d	ND	1568±16b
**SN8**	ND	33±3b	190±5	43±3b	260±20b	59420±106b	180±5b	374±6ab	ND	180±5b	1154±50a	233±8e	33±3c	ND
**SN9**	55±5b	ND	387±8	19±2f	ND	NDi	214±5a	601±12a	ND	226±5a	901±50c	406±9b	55±5b	ND

Means followed by the Same letter are not significantly different (p ≤ 0.05; Kruskal–Wallis test). Statistical analysis were performed for each column varying the node position (vertically per column) and letters were attributed according to the significance of the node position. S: stem, N: node and the number indicates the node number starting from the top (1).

Table S17. Isoprenoid cytokinin conjugates (iPA: isopentenyladenosine, DHZN7G: dihydrozeatin 7-glucoside, ZN7G: zeatin 7-glucoside, DHZN9G: dihydrozeatin 9-glucoside, ZN9G: zeatin 9-glucoside, DHZOG: dihydrozeatin O-glucoside, ZOG: zeatin O-glucoside, iP7G: isopentenyladenosine 7-glucoside and iP9G: isopentenyladenosine 9-glucoside) in shoots originated from the apical section (SAS) cultivated at density of 24 (D24). LN refers to leaves of node and the numbers from 1 to 11 refer to the number of the node starting from 1 the top node.

	**iPA**	**DHZN7G**	**ZN7G**	**DHZN9G**	**ZN9G**	**DHZOG**	**ZOG**	**iP7G**	**iP9G**	**Isoprenoid Free bases**	**Isoprenoid O-glucosides**	**Isoprenoid N-glucosides**
**LN1**	56±2c	ND	8553±50l	29±7a	78±3f	156±5g	445±5d	85±15abc	1351±50g	56±2c	601±10d	10096±26k

**LN2**	48±3d	45±5b	14003±100j	ND	118±3ef	116±5g	478±8c	110±20a	1801±50d	48±3d	593±13d	16077±24i
**LN3**	48±2d	115±5a	16874±75i	ND	63±3g	160±10g	541±10b	59±10cde	1459±52f	48±2d	701±20c	18570±14h
**LN4**	30±2g	ND	11878±75k	ND	ND	268±8d	ND	52±13de	1351±50g	30±2g	268±8e	13281±14j
**LN5**	39±2e	ND	28513±102f	ND	165±5c	268±8e	ND	90±20ab	2351±50a	39±2e	268±8e	31118±34e
**LN6**	35±2f	ND	25547±50g	ND	135±5d	211±10f	ND	54±15de	1759±53de	35±2f	211±10e	27495±22f
**LN7**	38±2ef	ND	20350±50h	ND	55±5g	566±15c	161±10e	45±15def	1905±51c	38±2ef	727±25c	22355±22g
**LN8**	59±2c	ND	32618±105c	ND	234±5a	727±25b	1052±50a	20±10f	2201±100b	59±2c	1779±75a	35074±36c
**LN9**	77±3b	ND	34510±101b	ND	185±5b	1376±75a	ND	73±18bcd	1682±76e	77±3b	1376±75b	36450±30b
**LN10**	38±3ef	ND	31570±75e	ND	160±10c	ND	ND	35±15ef	1253±50g	38±3ef	ND	33018±4d
**LN11**	83±3a	ND	44677±75a	ND	ND	ND	ND	44±15def	1057±51h	83±3a	ND	45778±17a

Means followed by the Same letter are not significantly different ($p \leq 0.05$; Kruskal–Wallis test). Statistical analysis were performed for each column varying the node position (vertically per column) and letters were attributed according to the significance of the node position. L: leaves, N: node and the number indicates the node number starting from the top (1).

Table S18. Isoprenoid cytokinin conjugates (DHZR : dihydrozeatin riboside, , tZR: trans-zeatin Riboside, tZ: trans-zeatin, iPA: isopentenyladenosine, DHZN7G: dihydrozeatin 7-glucoside, ZN7G: zeatin 7-glucoside, DHZN9G: dihydrozeatin 9-glucoside, ZN9G: zeatin 9-glucoside, ZOG: zeatin O-glucoside, iP7G: isopentenyladenosine 7-glucoside and iP9G: isopentenyladenosine 9-glucoside) in shoots originated from the apical section (SAS) cultivated at density of 24 (D24). SN refers to stems of node and the numbers from 1 to 12 refer to the number of the node starting from 1 the top node.

	**DHZR**	**tZR**	**tZ**	**iPA**	**DHZN7G**	**ZN7G**	**DHZN9G**	**ZN9G**	**ZOG**	**iP7G**	**iP9G**	**Isoprenoid Free bases**	**Isoprenoid ribosides**	**Isoprenoid O-glucosides**	**Isoprenoid N-glucosides**
**SN1**	ND	ND	124±6b	78±2g	ND	8987±103k	133±3a	ND	ND	545±51b	2047±50b	202±4c	ND	ND	11711±37k
**SN2**	ND	ND	ND	159±4b	165±5d	33140±128e	ND	108±3h	580±10c	264±15c	2382±28a	159±4d	ND	580±10c	36059±86e
**SN3**	ND	51±3b	ND	88±3f	68±4e	30474±75f	ND	143±3f	121±10d	121±10a	2020±26b	88±3g	51±3b	121±10d	32826±35f
**SN4**	ND	82±4a	74±1c	169±4a	ND	38470±75c	42±3e	215±5c	ND	150±10de	1625±25c	243±5b	82±4a	ND	40502±39c
**SN5**	ND	13±2e	ND	127±3c	ND	14207±101i	ND	187±3d	ND	160±10de	1428±26d	127±3e	13±2f	ND	15982±64i
**SN6**	12±3b	26±3c	ND	72±2	70±5e	22081±76h	88±3c	152±3ef	ND	185±15d	1428±26d	72±2h	38±0c	ND	24004±33h
**SN7**	15±5b	ND	146±2a	115±5d	185±5b	54602±100a	50±5d	320±5a	1976±25a	156±15de	1321±26e	261±7a	15±5f	1976±25a	56635±55a
**SN8**	ND	ND	71±1c	83±2f	275±5a	38205±100d	117±8b	222±3b	1038±8b	150±10de	849±50f	154±3d	ND	1038±8b	39818±40d
**SN9**	25±5a	ND	ND	66±3i	ND	23504±100g	ND	210±5c	ND	85±15f	825±25f	66±3h	25±5d	ND	24624±55g
**SN10**	ND	ND	ND	100±5e	ND	12976±75j	ND	ND	ND	272±25c	747±50g	100±5f	ND	ND	13995±9j

**SN11**	3±0c	17±2d	ND	102±3e	175±5c	45109±101b	ND	133±3g	ND	140±20e	1382±28d	102±3f	20±2e	ND	46939±55b
**SN12**	ND	ND	ND	51±2j	ND	8446±51l	ND	52±3i	ND	80±20f	1278±25e	51±2i	ND	ND	9856±4l

Means followed by the Same letter are not significantly different ($p \leq 0.05$; Kruskal–Wallis test). Statistical analysis were performed for each column varying the node position (vertically per column) and letters were attributed according to the significance of the node position. S: stem, N: node and the number indicates the node number starting from the top (1).

Table S19. Isoprenoid cytokinin conjugates (iPA: isopentenyladenosine, DHZN7G: dihydrozeatin 7-glucoside, ZN7G: zeatin 7-glucoside, DHZN9G: dihydrozeatin 9-glucoside, ZN9G: zeatin 9-glucoside, DHZOG: dihydrozeatin O-glucoside, ZOG: zeatin O-glucoside, iP7G: isopentenyladenosine 7-glucoside and iP9G: isopentenyladenosine 9-glucoside) in shoots originated from the middle section (SMS) cultivated at density of 24 (D24). LN refers to leaves of node and the numbers from 1 to 13 refer to the number of the node starting from 1 the top node.

	**iPA**	**DHZN7G**	**ZN7G**	**DHZN9G**	**ZN9G**	**DHZOG**	**ZOG**	**iP7G**	**iP9G**	**Isoprenoid Free bases**	**Isoprenoid O-glucosides**	**Isoprenoid N-glucosides**
**LN1**	ND	ND	3960±53l	24±1ef	35±5g	68±3f	135±5f	53±8efg	856±51h	ND	203±8g	4927±30l
**LN2**	ND	ND	3954±51m	17±1f	17±3h	93±3d	ND	35±5fgh	880±76h	ND	93±3h	4903±35m
**LN3**	ND	ND	4545±51k	13±1f	32±3g	90±5d	ND	26±10h	1421±26fg	ND	90±5h	6037±20k
**LN4**	9±1c	ND	5543±51j	12±1f	15±5h	177±8a	106±5g	57±8ef	2046±51c	9±1c	283±13d	7673±23i
**LN5**	ND	ND	5747±50i	ND	ND	83±3e	156±5e	32±10gh	1498±50f	ND	239±8e	7276±12j
**LN6**	ND	73±5b	9087±102h	34±1de	90±5e	60±5g	162±3d	57±8ef	1950±50d	ND	222±8f	11291±48h
**LN7**	7±2d	ND	15132±126g	20±1f	ND	108±8c	315±5b	48±8efgh	2197±50a	7±2d	423±13b	17397±69g
**LN8**	ND	ND	21652±50e	38±2d	62±3f	153±8b	186±5c	46±15efgh	2022±26cd	ND	339±13c	23819±9e
**LN9**	23±1a	ND	29062±54c	ND	148±3b	ND	ND	90±20cd	2152±50ab	23±1a	ND	31451±33c
**LN10**	14±2b	355±5a	49924±109a	73±3c	200±5a	ND	504±5a	116±15ab	2099±50bc	14±2b	504±5a	52767±51a
**LN11**	ND	ND	38002±100b	326±15b	115±5d	ND	ND	126±15a	1798±50e	ND	ND	40366±45b
**LN12**	ND	ND	23982±76d	650±20a	150±5b	ND	ND	100±20bc	1381±76g	ND	ND	26263±23d
**LN13**	ND	ND	18230±125f	64±2c	124±5c	ND	ND	70±20de	2149±50ab	ND	ND	20637±52f

Means followed by the Same letter are not significantly different ($p \leq 0.05$; Kruskal–Wallis test). Statistical analysis were performed for each column varying the node position (vertically per column) and letters were attributed according to the significance of the node position. L: leaves, N: node and the number indicates the node number starting from the top (1).

Table S20. Isoprenoid cytokinin conjugates (DHZR: dihydrozeatin riboside, tZR: trans-zeatin Riboside, iPA: isopentenyladenosine, DHZN7G: dihydrozeatin 7-glucoside, ZN9G: zeatin 9-glucoside, DHZOG: dihydrozeatin O-glucoside, ZOG: zeatin O-glucoside, iP7G: isopentenyladenosine 7-glucoside and iP9G: isopentenyladenosine 9-

glucoside) in shoots originated from the middle section (SMS) cultivated at density of 24 (D24). LN refers to leaves of node and the numbers from 1 to 13 refer to the number of the node starting from 1 the top node.

	DHZR	tZR	iPA	DHZN7G	ZN7G	DHZN9G	ZN9G	ZOG	iP7G	iP9G	Isoprenoid Free bases	Isoprenoid ribosides	Isoprenoid O-glucosides	Isoprenoid N-glucosides
**SN1**	26±6a	68±4a	80±2a	ND	4354±50l	ND	38±3i	ND	387±35a	1755±51b	80±2a	94±10a	ND	6533±42l
**SN2**	ND	22±3c	63±3b	ND	13477±75i	48±1h	113±3f	ND	166±15c	1925±25a	63±3b	22±3c	ND	15728±33i
**SN3**	ND	49±4b	45±2cd	204±4c	20077±75f	101±1d	125±5e	ND	132±10d	1651±50c	45±2cd	49±4b	ND	22289±10f
**SN4**	ND	ND	29±2f	ND	14979±75h	64±1f	ND	678±19c	131±10d	1649±50c	29±2f	ND	678±19c	16822±17h
**SN5**	ND	ND	ND	ND	9145±51j	41±1i	65±5g	318±8e	51±10e	1949±50a	NDa	ND	318±8e	11252±16j
**SN6**	ND	ND	22±2g	114±4d	32682±76e	53±1g	155±5d	ND	121±10d	1548±50d	22±2f	ND	ND	34673±13e
**SN7**	ND	ND	28±2f	ND	5650±50k	47±1h	52±3h	296±5f	121±10d	1553±50d	28±2g	ND	296±5f	7423±13k
**SN8**	ND	ND	46±2c	ND	4248±50l	34±1k	65±5g	ND	135±15d	1173±25f	46±2c	ND	ND	5655±9m
**SN9**	ND	ND	32±2e	ND	15481±76g	38±1j	130±5e	ND	186±15c	1024±25g	32±2e	ND	ND	16859±33g
**SN10**	ND	ND	33±2e	260±5a	39718±126c	77±1e	165±5c	ND	231±10b	974±25g	33±2e	ND	ND	41425±82c
**SN11**	ND	ND	43±2d	235±5b	48223±125a	152±3c	288±8b	1525±25a	235±15b	1327±25e	43±2d	ND	1525±25a	50460±75a
**SN12**	ND	ND	ND	ND	35630±125d	370±5a	554±5a	1277±8b	180±20c	690±36h	ND	ND	1277±8b	37424±72d
**SN13**	ND	ND	ND	233±3b	44937±127b	179±1b	170±5c	618±16d	61±10e	527±25i	ND	ND	618±16d	46107±88b

Means followed by the Same letter are not significantly different ($p \leq 0.05$; Kruskal–Wallis test). Statistical analysis were performed for each column varying the node position (vertically per column) and letters were attributed according to the significance of the node position. S: stem, N: node and the number indicates the node number starting from the top (1).

Table S21. Isoprenoid cytokinin conjugates (tZR: trans-zeatin Riboside, iPA: isopentenyladenosine, ZN7G: zeatin 7-glucoside DHZN9G: dihydrozeatin 9-glucoside, ZN9G: zeatin 9-glucoside, DHZOG: dihydrozeatin O-glucoside, ZOG: zeatin O-glucoside, iP7G: isopentenyladenosine 7-glucoside and iP9G: isopentenyladenosine 9-glucoside) in shoots originated from the apical section (SAS) cultivated at density of 30 (D30). LN refers to leaves of node and the numbers from 1 to 11 refer to the number of the node starting from 1 the top node.

	tZR	iPA	ZN7G	DHZN9G	ZN9G	DHZOG	ZOG	iP7G	iP9G	Isoprenoid Free bases	Isoprenoid ribosides	Isoprenoid O-glucosides	Isoprenoid N-glucosides
**LN1**	ND	ND	1943±52k	13±1d	ND	ND	ND	17±8e	827±25i	ND	ND	ND	2801±21k
**LN2**	ND	ND	3261±54j	ND	ND	95±2c	116±5b	ND	1055±51g	ND	ND	211±4a	4316±27j
**LN3**	ND	ND	5606±101i	125±5a	ND	ND	ND	ND	1120±26fg	ND	ND	ND	6852±80i
**LN4**	ND	21±2e	11310±101h	44±1c	58±3e	123±9b	ND	50±5d	1679±26e	21±2e	ND	123±9b	13139±72h
**LN5**	ND	ND	24133±126e	ND	ND	ND	125±5a	44±5d	2121±26a	ND	ND	125±5b	26298±95e
**LN6**	ND	ND	23511±102f	ND	70±5d	206±4a	ND	55±5d	1822±26cd	ND	ND	206±4a	25457±66f

**LN7**	ND	40±2d	25303±100c	ND	80±5c	ND	ND	78±8c	2044±51b	40±2d	ND	ND	27505±38c
**LN8**	ND	52±2c	34066±76b	ND	211±10b	ND	ND	102±8b	1753±50d	52±2c	ND	ND	36133±11b
**LN9**	ND	70±2a	36932±126a	ND	ND	ND	ND	80±10c	1851±50c	70±2a	ND	ND	38863±66a
**LN10**	ND	62±2b	24922±107d	120±5b	231±10a	ND	ND	130±10a	1147±50f	62±2b	ND	ND	26550±49d
**LN11**	141±6 a	ND	15702±100g	ND	ND	ND	ND	45±15d	950±50h	ND	141±6a	ND	16697±35g

Means followed by the Same letter are not significantly different ($p \leq 0.05$; Kruskal–Wallis test). Statistical analysis were performed for each column varying the node position (vertically per column) and letters were attributed according to the significance of the node position. L: leaves, N: node and the number indicates the node number starting from the top (1).

Table S22. Isoprenoid cytokinin conjugates (tZR: trans-zeatin Riboside, tZ: trans-zeatin, iPA: isopentenyladenosine, DHZN7G: dihydrozeatin 7-glucoside, ZN7G: zeatin 7-glucoside DHZN9G: dihydrozeatin 9-glucoside, ZN9G: zeatin 9-glucoside, ZOG: zeatin O-glucoside, iP7G: isopentenyladenosine 7-glucoside and iP9G: isopentenyladenosine 9-glucoside and ZROG: zeatin riboside O-glucoside) in shoots originated from the apical section (SAS) cultivated at density of 30 (D30). SN refers to stems of node and the numbers from 1 to 13 refer to the number of the node starting from 1 the top node.

	**tZR**	**tZ**	**iPA**	**DHZN7G**	**ZN7G**	**DHZN9G**	**ZN9G**	**ZOG**	**iP7G**	**iP9G**	**ZROG**	**Isoprenoid Free bases**	**Isoprenoid ribosides**	**Isoprenoid O-glucosides**	**Isoprenoid N-glucoside**
**SN1**	ND	ND	ND	ND	2300±100k	283±3a	5±0k	120±5f	221±10a	1849±50d	ND	ND	ND	120±5g	4657±42j
**SN2**	ND	ND	ND	ND	3805±100j	78±2g	ND	ND	90±5e	2277±25a	ND	ND	ND	ND	6250±72i
**SN3**	ND	ND	51±2f	ND	3746±150j	112±1d	31±1j	ND	40±5g	2147±50b	ND	51±2def	ND	ND	6076±96i
**SN4**	ND	ND	47±2g	ND	4983±202i	47±1i	47±3i	ND	43±3g	2027±25c	ND	47±2ef	ND	ND	7147±172i
**SN5**	ND	ND	65±2d	ND	10017±501g	82±1f	95±5g	655±5d	135±5bc	1781±27e	ND	65±2c	ND	655±5e	12110±468g
**SN6**	ND	ND	42±2h	ND	8057±151h	27±1j	77±3h	ND	70±5f	1319±27i	ND	42±2f	ND	ND	9551±119h
**SN7**	21±3a	ND	49±2fg	ND	10204±754g	49±1i	80±5h	518±16e	140±10b	1550±50g	ND	49±2def	21±3a	518±16f	12023±690g
**SN8**	ND	ND	71±2c	ND	39502±500c	93±1e	310±10c	ND	230±10a	1019±27k	ND	71±2bc	ND	ND	41155±456c
**SN9**	ND	ND	64±2d	ND	27756±227d	ND	220±10d	2280±26a	128±8bc	752±50l	718±50c	64±2c	ND	2997±26a	28857±160d
**SN10**	ND	ND	ND	ND	27266±251d	ND	133±3f	ND	74±5f	1179±26j	ND	ND	ND	ND	28652±220d
**SN11**	ND	ND	76±2a	ND	14473±502f	138±2c	ND	1211±10c	127±8bc	1455±51h	1534±34a	76±2b	ND	2745±44b	16193±446f
**SN12**	ND	282±18a	73±2b	155±15a	65445±1503a	149±1b	340±10a	ND	125±5d	1454±51h	ND	356±16a	ND	ND	67668±1424a
**SN13**	ND	ND	76±2a	ND	22275±751e	59±1h	151±10e	2028±26b	71±10f	1718±28ef	ND	76±2b	ND	2028±26c	24274±707e
**SN14**	ND	ND	56±2e	ND	42484±1500b	ND	326±15b	ND	45±5g	1699±50f	940±29b	56±2d	ND	940±29d	44554±1430b

Means followed by the Same letter are not significantly different (p ≤ 0.05; Kruskal–Wallis test). Statistical analysis were performed for each column varying the node position (vertically per column) and letters were attributed according to the significance of the node position. S: stem, N: node and the number indicates the node number starting from the top (1).

Table S23. Isoprenoid cytokinin conjugates (DHZR: dihydrozeatin riboside, iPA: isopentenyladenosine, ZN7G: zeatin 7-glucoside, DHZN9G: dihydrozeatin 9-glucoside, ZN9G: zeatin 9-glucoside, DHZOG: dihydrozeatin O-glucoside, ZOG: zeatin O-glucoside, iP7G: isopentenyladenosine 7-glucoside and iP9G: isopentenyladenosine 9-glucoside and ZROG: zeatin riboside O-glucoside) in shoots originated from the middle section (SMS) cultivated at density of 30 (D30). LN refers to leaves of node and the numbers from 1 to 13 refer to the number of the node starting from 1 the top node.

	**DHZR**	**iPA**	**ZN7G**	**DHZN9G**	**ZN9G**	**DHZOG**	**ZOG**	**iP7G**	**iP9G**	**ZROG**	**Isoprenoid Free bases**	**Isoprenoid ribosides**	**Isoprenoid O-glucosides**	**Isoprenoid N-glucosides**
**LN1**	ND	ND	2154±50k	ND	14±1j	ND	ND	57±3ef	1051±50fg	443±24a	ND	ND	443±24c	3276±9l
**LN2**	ND	ND	3816±104j	ND	ND	ND	ND	42±3g	802±50h	ND	ND	ND	ND	4660±57k
**LN3**	ND	ND	5652±150i	ND	52±3h	ND	ND	55±5ef	1101±50f	ND	ND	ND	ND	6860±93j
**LN4**	ND	ND	6314±103h	ND	ND	113±12b	92±3e	58±3ef	1805±51b	ND	ND	ND	205±10g	8176±58i
**LN5**	ND	ND	7053±150g	ND	37±3i	85±5c	57±3f	52±3fgh	1424±25d	ND	ND	ND	142±7h	8567±120h
**LN6**	ND	14±2c	19278±225f	ND	103±3f	156±41a	168±8d	66±5de	1425±25d	ND	14±2c	ND	325±33e	20871±193g
**LN7**	ND	10±2d	20213±258e	ND	175±5c	147±8a	248±8c	73±3cd	1498±50d	ND	10±2d	ND	395±15d	21958±204f
**LN8**	ND	17±2b	24531±475d	ND	190±5b	165±26a	ND	82±3c	1645±51c	ND	17±2b	ND	165±26h	26448±418d
**LN9**	ND	19±2b	29740±251b	ND	90±5g	121±16b	156±5d	121±10b	1601±50c	ND	19±2b	ND	277±11f	31552±185b
**LN10**	22±7a	65±4a	52246±250a	127±23b	320±10a	ND	1527±25a	356±15a	2005±51a	ND	65±4a	22±7a	1527±25a	55054±198a
**LN11**	ND	ND	24926±125d	ND	153±3d	ND	492±11b	30±10h	1279±75e	ND	ND	ND	492±11b	26387±38d
**LN12**	ND	ND	24509±500d	ND	140±5e	ND	ND	80±10c	996±50g	ND	ND	ND	ND	25726±435e
**LN13**	ND	ND	27944±82c	690±10a	ND	ND	ND	ND	877±75h	ND	ND	ND	ND	29512±39c

Means followed by the Same letter are not significantly different (p ≤ 0.05; Kruskal–Wallis test). Statistical analysis were performed for each column varying the node position (vertically per column) and letters were attributed according to the significance of the node position. L: leaves, N: node and the number indicates the node number starting from the top (1).

Table S24. Isoprenoid cytokinin conjugates (tZR: trans-zeatin Riboside, iPA: isopentenyladenosine, DHZN7G: dihydrozeatin 7-glucoside, ZN7G: zeatin 7-glucoside, DHZN9G: dihydrozeatin 9-glucoside, ZN9G: zeatin 9-glucoside, DHZOG: dihydrozeatin O-glucoside, ZOG: zeatin O-glucoside, iP7G: isopentenyladenosine 7-glucoside, iP9G: isopentenyladenosine 9-glucoside, ZROG: zeatin riboside O-glucoside and DHZROG: dihydrozeatin riboside O-glucoside) in shoots originated from the middle section (SMS) cultivated at density of 30 (D30). SN refers to stems of node and the numbers from 1 to 13 refer to the number of the node starting from 1 the top node.

	tZR	iPA	DHZN7G	ZN7G	DHZN9G	ZN9G	DHZOG	ZOG	iP7G	iP9G	ZROG	DHZROG	Isoprenoid Free bases	Isoprenoid ribosides	Isoprenoid O-glucosides	Isoprenoid N-glucosides
**SN1**	ND	33±2e	ND	10003±100g	ND	72±3h	ND	ND	346±15a	1727±25c	450±51b	ND	33±2e	ND	450±51e	12148±58g
**SN2**	20±3b	38±2d	ND	4045±51k	ND	36±1i	ND	ND	86±5f	2076±25b	427±19b	ND	38±2d	20±3b	427±19e	6243±20l
**SN3**	ND	18±2g	ND	7299±100j	ND	93±3f	ND	218±8f	136±5de	1655±51d	ND	ND	18±2g	ND	218±8g	9183±43j
**SN4**	ND	32±2e	45±5d	12425±75e	64±2d	85±5g	ND	302±3d	171±10c	2153±50a	ND	275±34a	32±2e	ND	578±36d	14943±11f
**SN5**	24±3a	78±3a	69±4c	10105±100g	ND	98±3f	ND	286±6e	138±8d	1530±26e	ND	ND	78±3a	24±3a	286±6f	11940±64h
**SN6**	ND	31±2e	ND	23162±102b	133±3a	208±3a	ND	811±10b	128±8de	1427±25f	ND	ND	31±2e	ND	811±10b	25058±72b
**SN7**	ND	52±2b	ND	16114±78c	ND	120±5d	ND	ND	120±10e	1555±51e	ND	ND	52±2b	ND	ND	17909±14d
**SN8**	ND	41±2c	127±8a	23074±75b	ND	156±5c	ND	771±10c	256±15b	928±25g	ND	ND	41±2c	ND	771±10c	24540±23c
**SN9**	ND	4±1h	ND	8001±100h	42±3f	73±3h	148±4a	87±3g	30±5g	451±50k	ND	ND	4±1h	ND	235±1g	8596±45k
**SN10**	ND	15±2g	ND	32859±128a	ND	173±3b	ND	951±10a	135±5de	728±26i	ND	ND	15±2g	ND	951±10a	33895±95a
**SN11**	ND	ND	ND	7707±101i	88±3c	75±5h	ND	ND	158±8c	525±25j	ND	ND	ND	ND	ND	8552±66k
**SN12**	ND	23±2f	101±6b	14939±102d	50±5e	110±5e	13±1b	ND	80±5f	851±50h	561±36a	ND	23±2f	ND	573±37d	16131±43e
**SN13**	ND	ND	ND	10658±129f	117±8b	82±3g	ND	ND	80±5f	210±10l	ND	ND	ND	ND	ND	11147±119i

Means followed by the Same letter are not significantly different ($p \leq 0.05$; Kruskal–Wallis test). Statistical analysis were performed for each column varying the node position (vertically per column) and letters were attributed according to the significance of the node position. S: stem, N: node and the number indicates the node number starting from the top (1).
